# Genetic Characterization of the O-Antigen and Development of a Molecular Serotyping Scheme for *Enterobacter cloacae*

**DOI:** 10.3389/fmicb.2020.00727

**Published:** 2020-04-28

**Authors:** Yayue Li, Junjie Huang, Xiaotong Wang, Cong Xu, Tao Han, Xi Guo

**Affiliations:** ^1^Key Laboratory of Molecular Microbiology and Technology, Ministry of Education, TEDA Institute of Biological Sciences and Biotechnology, Nankai University, Tianjin, China; ^2^The Third Central Hospital of Tianjin, Tianjin Key Laboratory of Extracorporeal Life Support for Critical Diseases, Tianjin Institute of Hepatobiliary Disease, Tianjin, China; ^3^Department of Vascular Surgery, Tianjin Hospital, Tianjin, China; ^4^Tianjin Children’s Hospital, Third Central Clinical College of Tianjin Medical University, Tianjin, China

**Keywords:** *Enterobacter cloacae*, serotype, O-antigen, gene cluster, multiplex PCR

## Abstract

*Enterobacter cloacae* is a well-characterized opportunistic pathogen that is closely associated with various nosocomial infections. The O-antigen, which is one of the most variable constituents on the cell surface, has been used widely and traditionally for serological classification of many gram-negative bacteria. *E. cloacae* is divided into 30 serotypes, based on its O-antigen diversity. In this study, by using genomic and comparative-genomic approaches, we analyzed the O-antigen gene clusters of 26 *E. cloacae* serotypes in depth. We also identified the sero-specific gene for each serotype and developed a multiplex polymerase chain reaction (PCR) method. The sensitivity of the assay was 0.1 ng for genomic DNA and 10^3^ colony forming units for pure cultures. The assay reliability was evaluated by double-blinded testing with 81 clinical strains. Furthermore, we established a valid, genome-based tool for *in silico* serotyping of *E. cloacae*. By screening 431 *E. cloacae* genomes deposited in GenBank, 304 were classified into current antigenic scheme, and 112 were allocated into 55 putative novel serotypes. Our results represent the first genetic basis of the O-antigen diversity and variation of *E. cloacae*, providing a rationale for studying the O-antigen associated evolution and pathogenesis of this bacterium. In addition, we extended the current serotyping system for *E. cloacae*, which is important for detection and epidemiological surveillance purposes for this important pathogen.

## Introduction

*Enterobacter cloacae*, which is ubiquitous in soil, water, and sewage, is a well-known human opportunistic pathogen that is frequently responsible for nosocomial infections contributing to bacteremia, endocarditis, septic arthritis, osteomyelitis, skin/soft tissue infections, and lower respiratory tract, urinary tract, and intra-abdominal infections (Fata et al., 1996; Mezzatesta et al., 2012; Davin-Regli and Pagès, 2015). *E. cloacae* has been implicated repeatedly as a nosocomial pathogen in neonatal units, and several outbreaks of *E. cloacae* infections have been reported (Marra et al., 2011; Qureshi et al., 2011; Pestourie et al., 2014). In recent decades, *E. cloacae* has emerged as the third most frequent and lethal Enterobacteriaceae species involved in bloodstream infections (Wang et al., 2017). Moreover, with the extensive use of broad-spectrum antibiotics over extended periods of time, the increasing prevalence of multidrug-resistant isolates in different populations has become a growing concern (Mezzatesta et al., 2012; Annavajhala et al., 2019).

Lipopolysaccharide (LPS), which is a hallmark structural entity, is essential for membrane stability and cell survival and is a key virulence determinant for many gram-negative bacterial species. LPS molecules are typically composed of three segments: lipid A anchoring LPS to the outer membrane; a core oligosaccharide, that is a non-repeating oligosaccharide commonly consisting of monosaccharides as such as heptose and keto-deoxyoctulosonate; and O-antigen (O-polysaccharide), which is a polymer of repeating oligosaccharide (O-units), each ranging from two to seven residues from a broad range sugars and their derivatives (Valvano, 2003; Merino et al., 2016). In many cases, the O-antigen contributes the most to cell-surface diversity in gram-negative bacteria, thus offering a selective advantage in its specific niche (Reeves, 1992; Wang et al., 2010), and is also an key virulence factor associated with bacterial pathogenesis (March et al., 2013; Sarkar et al., 2014; Caboni et al., 2015). In particular, considerable variation of the O-antigen composition provides a basis for serotyping schemes with many gram-negative bacteria, which has been recognized one of the most important cell constituents in typing strains, and a basic tool utilized in outbreak investigations and epidemiological survey (Kenyon et al., 2017; DebRoy et al., 2018; Guo et al., 2018; Qian et al., 2018).

The major genes for O-antigen synthesis are generally clustered at a chromosomal locus that maps between two housekeeping genes, namely O-antigen gene cluster (O-AGC). These genes are commonly classified into three main classes: nucleotide sugar precursor synthesis genes for sugars that are specific to the particular polysaccharide; glycosyltransferase genes that are associated with the O-unit assemblies and are specific for donor and acceptor sugars, and generate a specific linkage between them; and O-unit processing genes for O-unit translocation and polymerization. Furthermore, three different pathways are known for their involvement in O-antigen synthesis, which are generally named after the proteins involved: the Wzx/Wzy-dependent pathway, the ATP-binding cassette (ABC) transporter (Wzm/Wzt)-dependent pathway, and the synthase-dependent pathway (Keenleyside and Whitefield, 1996; Samuel and Reeves, 2003; Liu et al., 2008, 2014). All O-antigen biosynthesis pathways are initiated by the transfer of a sugar phosphate from an NDP-sugar to the carrier lipid, undecaprenyl phosphate (Und-P), forming Und-PP-sugar (Reeves and Wang, 2002). In the Wzx/Wzy pathway, sugars are transferred one by one from the respective sugar nucleotides to Und-PP-sugar by glycosyltransferases to form O-unit, then, the Und-PP-linked O-units are flipped by the flippase protein, Wzx, across the inner membrane to the periplasm, where the O-unit is polymerized by the polymerase protein, Wzy, to generate the polymer (Reeves and Wang, 2002). In the ABC transporter pathway, the O-antigen is synthesized directly on the Und-PP-sugar, and the translocation of the Und-PP linked O-antigen is carried out by an ABC transporter. The ABC transporter is typically composed of two transmembrane domains (Wzm) and two nucleotide binding domains (Wzt), with the former forming the translocation channel and the latter driving the transport cycle by hydrolyzing ATP (Cuthbertson et al., 2007; Whitfield and Trent, 2014). Following translocation and polymerization, the resultant O-antigen is then attached to the lipid A-core by the ligase, WaaL, to generate mature LPS molecules (Han et al., 2011; Ruan et al., 2012), and the LPS will be transported to the outer membrane by the Lpt pathway (Silhavy et al., 2010).

In Sakazaki and Namioka (1960) firstly reported on the serology of 170 *E. cloacae* strains, and 53 O-antigens and 56 H-antigens of *E. cloacae* were distinguished in agglutination tests. In 1983, an antigenic scheme comprising 28 heat-stable O-antigen types, which is still currently accepted, was developed by Gaston et al. (1983), followed by the subsequent addition of another two serotypes^1^.

The O-AGC of *E. cloacae* has been reported to be located between two housekeeping genes, *galF* and *gnd*, and shows perfect correlations with each O-antigen structure in several isolates (Filatov et al., 2014; Perepelov et al., 2014, 2015, 2016, 2017; Han et al., 2017). These previous studies shed clear genetic and evolutionary information regarding O-AGC of *E. cloacae*. However, the isolates used in those studies were not reference strains and their serotypes were not indicated. Here, we present a detailed analysis of the O-AGCs of 26 *E. cloacae* reference strains with available O-serotypes. Moreover, a sero-specific multiplex polymerase chain reaction (PCR) assay was developed, and its specificity and sensitivity were evaluated. We also screened the serotype distribution of 431 isolates with available genomes deposited in GenBank, using the sero-specific genes characterized in this study, and 55 putative novel gene clusters were characterized by us, extending dramatically the current antigenic scheme of *E. cloacae*. Our current work provides a valuable framework for further assessing the evolution of *E. cloacae*, and the developed molecular-serotyping assay gives a potential for molecular diagnostics and epidemiological surveillance of this important pathogen.

## Materials and Methods

### Bacterial Strains and Genomic-DNA Extraction

Details for all bacterial strains used in this study are summarized in Table 1. These strains included 26 reference strains with known serotypes and 81 clinical isolates. Sixteen other strains from eight species within the Enterobacteriaceae family were used to assess the specificity of our multiplex PCR. All strains were grown overnight in Luria-Bertani medium at 37°C with shaking, and genomic DNA was extracted using the TIANamp Bacteria DNA Kit (Tiangen, Beijing, China) according to the manufacturer’s protocol.

**TABLE 1:** Bacterial strains used in this study.

**Species**	**Strain No. (source^a^)**	**Total**
***E. CLOACA*E REFERENCE STRAINS**		
O1	11570 (NCTC)	1
O3	11572 (NCTC)	1
O4	11573 (NCTC)	1
O5	11574 (NCTC)	1
O6	11575 (NCTC)	1
O7	11576 (NCTC)	1
O8	11577 (NCTC)	1
O9	11578 (NCTC)	1
O10	11579 (NCTC)	1
O11	11580 (NCTC)	1
O12	11581 (NCTC)	1
O13	11582 (NCTC)	1
O14	11583 (NCTC)	1
O15	11584 (NCTC)	1
O16	11585 (NCTC)	1
O17	11586 (NCTC)	1
O18	11587 (NCTC)	1
O19	11588 (NCTC)	1
O20	11589 (NCTC)	1
O21	11590 (NCTC)	1
O22	11591 (NCTC)	1
O23	11592 (NCTC)	1
O24	11593 (NCTC)	1
O26	11595 (NCTC)	1
O27	11596 (NCTC)	1
O30	11932 (NCTC)	1
*E. cloaca*e clinical isolates		81
**Strains From Other Species (*n* = 16)**		
*Enterobacter aerogenes*	12058 (DSM), 30053 (DSM), 51697 (ATCC), 35029 (ATCC)	4
*Enterobacter sakazakii*	3460 (CCM), 3461 (CCM), 24133 (JCM)	3
*Klebsiella pneumonia*	46102 (CMCC), 46112 (CMCC) 46105 (ATCC)	3
*Yersinia enterocolitica*	51872 (ATCC), 35669 (ATCC)	2
*Proteus vulgaris*	49990 (ATCC)	1
*Salmonella typhi*	50071-9 (CMCC)	1
*Salmonella paratyphi*	50017-15b (CMCC)	1
*Shigella flexneri 2a*	700930 (ATCC)	1

**^*a*^NCTC, National Collection of Type Cultures, United Kingdom; ATCC, American Type Culture Collection, USA; DSM, German National Resource Centre for Biological Material; CCM, Czech Collection of microorganisms; CMCC, National Centerfor Medical Culture Collections; JCM, Japan Collection of Microorganisms.**

### Sequencing and Bioinformatics Analysis

Whole-genome sequencing (WGS) of 26 *E. cloacae* reference strains was performed with Solexa paired-end sequencing technology. In general, the genomic DNA were sheared, polished, and prepared using the Illumina Sample Preparation Kit. Genomic libraries were constructed containing 500 bp paired-end inserts, and sequencing was then performed via Solexa sequencing technology (Illumina, Inc.) for ∼100-fold coverage. The reads obtained were assembled using the *de novo* genome-assembly program, Velvet, to generate a multi-contig draft genome. Gaps within the O-AGCs were closed by directed PCR, and the products were sequenced using BigDye terminator chemistry on ABI 3730 capillary sequencers.

The Artemis program (Rutherford et al., 2000) was used for annotation and the lockMaker program (Henikoff et al., 1995) was used to identify conserved motifs. The BLAST and PSI-BLAST programs (Altschul et al., 1997) were used to search available databases, including GenBank^2^ and the Pfam protein motif databases^3^. The TMHMM v2.0 analysis program^4^ was used to identify potential transmembrane domains within protein sequences. O-AGC sequences, between *galF* and *gnd* genes of each strain, were retrieved from the genomes for further analysis.

### Development of a Multiplex PCR Assay

All sero-specific primers were designed based on the *wzy* gene sequences determined in this study, except for serotype O23, for which the *wzt* gene was targeted (Table 2). The specificity of each individual primer pair was confirmed using the BLAST program and was subsequently validated by a single PCR amplification, using the strains listed in Table 1. Each PCR was performed in a 25 μl reaction mixture containing 50 ng genomic DNA, 1 × Goldstar PCR buffer, 0.04 mM deoxynucleoside triphosphates, 0.1 μM each primer, and 1 unit Goldstar DNA polymerase. The PCR program used was as follows: denaturation at 95° C for 10 min; 30 cycles of denaturation at 95° C for 30 s, annealing at 55° C for 30 s, and extension at 72° C for 1 min; followed by a final extension at 72° C for 5 min.

**TABLE 2:** Primers used in this study.

**Group**	**Serotype**	**Targeted gene**	**Froward primer (5′–3′)**	**Reverse primer (5′–3′)**	**Amplicon size (bp)**
1	O1	*wzy*	ATTTTCTGAACGAGGTCTTGATA	AAGATAAGTGGTTAGCACGAGG	211
	O3	*wzy*	TGATGGCTATTCTGCTCTGG	TTCCCAACCACCGTAGCC	311
	O4	*wzy*	TGCTCCTGCTGAAGGTGTC	ATGGCAGTTCCTATTATTCCG	411
	O5	*wzy*	AGTTTCGTTTGCTTGGCTC	TTCTCCCACGGTCCTCTGT	523
	O6	*wzy*	TATGAAAACAATCGGTTACGC	GCCAATCTATACCAACCAACAT	657
	O7	*wzy*	CTGTTCGTCTTCTTGTTAATCGT	CAAATAATCTAATGTGTATCCCCTG	714
	O8	*wzy*	ATCTCCCTGTATTACTTTTATTTAGC	GCTGAGGTAATTAGAGGTCTAACAG	850
	O9/O10/O11	*wzy*	AGCGTTTATATATTTCCTGCTTACT	GCTATCCCATTAGACACGCT	1008
2	O12	*wzy*	TATGCCTGTATGTTGTCTTTGC	TAACTAATACCAAAAAGCGGC	400
	O13	*wzy*	CTATCGCAGGTTTTAGACCCA	AAAGGTATTGTTAAAAATCCGAAT	841
	O14	*wzy*	GTCACTTTATTTTGTTGGTTTGG	GTTCCGTGATCGTTAAGACAA	215
	O15	*wzy*	TTTTGGCAGGAAGTCGTAAG	CGCTCTACCAAAGAAATTCAG	308
	O16	*wzy*	GTGCTTTGCGATAATACCTGA	ACCCGCAGTAACATAGACATAAA	475
	O17	*wzy*	ATGGCTTTCTCGTTTAGTTCG	GACTTCCCCACCACTCAACT	651
	O18	*wzy*	TTCTGGCTGTGATGTTTTCG	CAGCGTTAAATCCAATCAAGAC	1137
	O19	*wzy*	TGGATACAGGGTATTCCGCTA	AATCGCAAACTCATTGAAGAAG	738
3	O20	*wzy*	AACGACGCTATGTTTCTTTTG	CCGAACCACATAACCACAAA	537
	O21	*wzy*	CATTTATTCCATTTTTAAGCTCTG	CGCATAAACTTTCTCCCGA	938
	O22	*wzy*	GAGATTCGGAAACGGACTTG	TATATCACAATGTTTATCACTGCC	342
	O23	*wzt*	GGCTCCATTTCTTGTCTGCT	TATCCGAGTCAAGATGAGCAC	241
	O24	*wzy*	TATTTGTATGCGTGCCAGAAG	ACTCAGATAGTATATTACCCGCAA	441
	O26	*wzy*	GCATCGGTCAATCCTCAAG	ACAAGCCAGCACATCCAAC	206
	O27	*wzy*	ATGGTTTACCGATGTCTACTGG	TTATACACCTTTTAATCGCCTATTA	832
	O30	*wzy*	GTAATTGATGGTTTATGGCGTT	AGTGAGCAAAGGAATGAGAAAGT	722

The multiplex PCR assay consisted of three groups (Table 2). Group 1 comprised serotypes O1, O3, O4, O5, O6, O7, O8, and O9/10/11; group 2 comprised serotypes O12, O13, O14, O15 O16, O17, O18, and O19; and group 3 comprised serotypes O20, O21, O22, O23, O24, O26, O27, and O30. The PCR mixtures and the PCR program used were the same as those for the singleplex PCR amplifications.

### Construction of an *in silico* Serotyping Program

A Python script was constructed for *E. cloacae* serotyping using genomic data (Supplementary Data S1). Generally, a database was first generated based on the sero-specific genes characterized and tested in this study, i.e., the *wzy* genes for 25 of the 26 serotypes and the *wzt* gene for serotype O23. Next, genomic assemblies were employed to a BLASTn search against the database with an identity cutoff of >99%. The script outputs contained the best-matching genes via BLASTn analysis, as well as the identity level between sero-specific gene(s) and homologous genes(s) in the query genome, which enabled determination of the exact serotype.

### Nucleotide Sequence Accession Number

The DNA sequences of the O-AGCs from all 26 *E. cloacae* reference strains were deposited in GenBank database under accession numbers MK595714 to MK595739.

## Results

### Analysis of the *E. cloacae* O-AGCs

**FIGURE 1:**
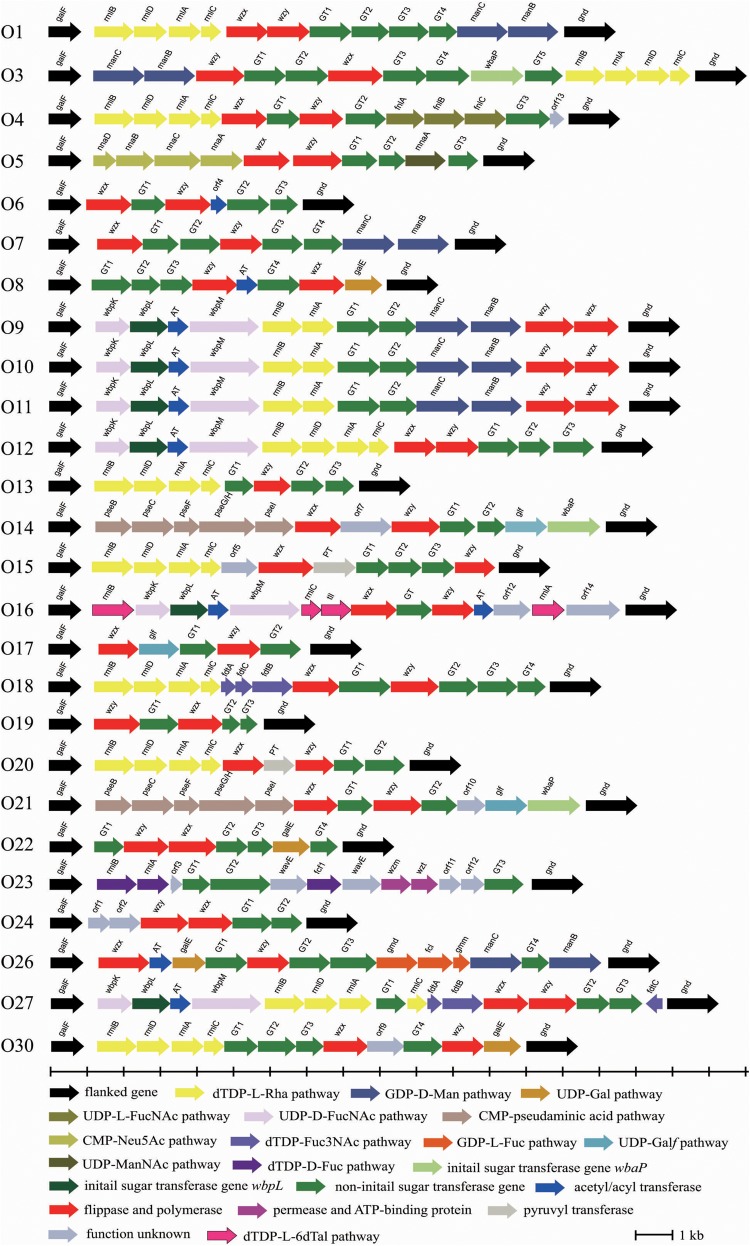
Schematic diagram of the O-AGCs identified from the 26 *E. cloacae* reference strains. Genes are represented by arrows and colored according to the gene key at the bottom with gene names indicated above each arrow.

Twenty-six O-AGCs of *E. cloacae* reference strains collected from National Collection of Type Cultures, United Kingdom (NCTC) were obtained via genome sequencing. All O-AGCs are located between two housekeeping genes, *galF* and *gnd*, and range from 4,473 to 16,323 bp, with all genes being transcribed from *galF* and *gnd* (except the *fdtC* gene of O27) and five to 16 open reading frames (ORFs) being encoded. Generally, the main three classes of genes within the O-AGC were annotated in each serotype. In addition, several pyruvyl transferases, acetyl transferases, and hypothetical protein encoding genes were also assigned for individual strains. Figure 1 shows a schematic representation of all 26 O-AGCs, and the characteristics of all ORFs within each O-AGC are summarized in Supplementary Table S1.

**FIGURE 2:**
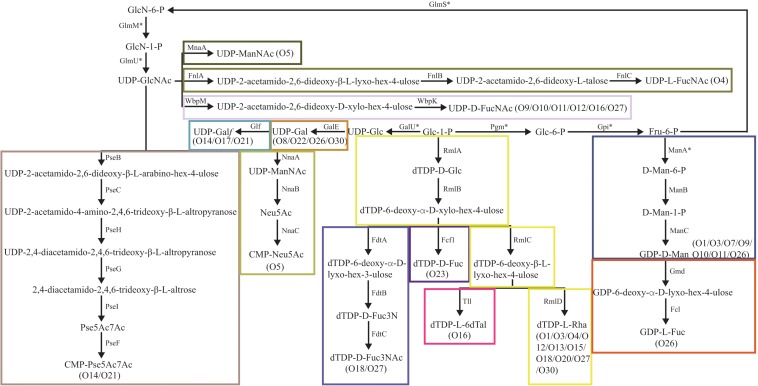
Biosynthesis pathways for the putative sugars in the *E. cloacae* O-antigens. MnaA, UDP-N-acetylglucosamine-2-epimerase (Campbell et al., 2000); FnlA, 4,6-dehydratase, 3- and 5-epimerase; FnlB, reductase; FnlC, C-2 epimerase (Kneidinger et al., 2003); GalE, UDP-glucose-4-epimerase (Samuel and Reeves, 2003); Glf, UDP-galactopyranose mutase (Nassau et al., 1996); GalU, UTP-glucose-1-phosphate uridylyltransferase (Bonofiglio et al., 2005); GlmU, UDP-N-acetyl-glucosamine pyrophosphorylase (Mengin-Lecreulx and van Heijenoort, 1993); GlmM, phosphoglucosamine mutase (Mengin-Lecreulx and van Heijenoort, 1996); GlmS, L-Glutamine:D-fructose-6-phosphate amidotransferase (Badet et al., 1987); Pgm, phosphoglucomutase (Lu and Kleckner, 1994); Gpi, Glucose-6-phosphate isomerase (Winkler, 1970); NnaA, UDP-N-acetylglucosamine-2-epimerase; NnaB, N-acetylneuraminic acid synthetase; NnaC, CMP-N-acetylneuraminic acid synthetase (Annunziato et al., 1995); RmlA, glucose-1-phosphate thymidylyltransferase (Zuccotti et al., 2001); RmlB, dTDP-D-glucose 4,6-dehydratase (Allard et al., 2001); RmlC, dTDP-4-keto-6-deoxy-D-glucose 3,5-epimerase (Giraud et al., 1999a); RmlD, dTDP-6-deoxy-L-mannose-dehydrogenase (Giraud et al., 1999b); ManA, phosphomannose isomerase; ManB, phosphomannomutase; ManC, mannose-1-phosphate guanylyltransferase (Samuel and Reeves, 2003); Gmd, GDP-mannose-4,6-dehydratase (Somoza et al., 2000; Kneidinger et al., 2001); Fcl, GDP-L-fucose synthetase (Rosano et al., 2000); FdtA, dTDP-6-deoxy-hex-4-ulose isomerase; FdtB, dTDP-6-deoxy-D-xylo-hex-3-ulose aminase; FdtC, dTDP-D-Fuc3N acetylase (Pfoestl et al., 2003); WbpM, UDP-D-GlcNAc 4,6-dehydratase; WbpK, 4-reductase (King et al., 2009); PseB, C6 dehydratase/C5 epimerase; PseC, aminotransferase; PseH, N-acetyltransferase; PseG, nucleotidase; PseI, condensase; PseF, cytidylyltransferase (Schoenhofen et al., 2006); Fcf1, dTDP-6-deoxy-D-xylo-hex-4-ulopyranose reductase (Wang et al., 2008); Tll, dTDP-6-deoxy-L-lyxo-4-hexulose reductase (Nakano et al., 2000).

Some normal sugars, including D-GlcNAc, D-Glc, D-GlcA, and D-GalA, are also found in other structures in the Enterobacteriaceae family and the biosynthesis genes are normally found at various loci outside the O-AGCs. Here, the biosynthesis pathway of 13 rare occurring sugars was proposed based on the occurrence of their corresponding nucleotide sugar precursor synthesis genes (Figure 2).

The O-antigen synthesis pathway is initiated by transfer of a sugar phosphate from an NDP-sugar to Und-P. In most *E. coli* and *Shigella* strains, and in a high proportion of *Salmonella* strains, WecA, encoded by *wecA* gene of the enterobacterial common antigen gene cluster, mediates this step by transferring UDP-GlcNAc to Und-P (Alexander and Valvano, 1994; Rick et al., 1994; Al-Dabbagh et al., 2016). Among the 26 serotypes, 17 (65%) possess no initial transferase (IT) gene, which probably means that O-antigen synthesis is mediated by WecA in those serotypes. Indeed, we discovered a homolog of the *wecA* gene by screening each genome for all of them (data not shown). In serotypes O9 to O12, O16, and O27, the homologs of *wbpL* gene were found, whose product has been identified as an IT and transferred UDP-D-FucNAc to Und-P to initiate O-antigen synthesis in *Pesudomonas aeruginosa* (Rocchetta et al., 1998). We also assigned *wbpM* and *wbpK* genes in these serotypes, whose products were characterized collectively in terms of UDP-D-FucNAc formation in *P. aeruginosa* (King et al., 2009). Thus, we deduced that the initial sugar of the O-antigens of O9 to O12, O16, and O27 is very likely D-FucNAc. Another IT gene, *wbaP*, was assigned to O3, O14, and O21. WbaP was previously identified as an IT that transfer Gal-1-P to Und-P to initiate O-antigen synthesis (Reeves et al., 2013). We therefore propose that the initial sugar of the O-antigens of these three serotypes should be D-Gal.

Three different pathways have been reported for O-antigen synthesis. For *E. cloacae*, 96% (25 of 26 serotypes) O-AGCs contain *wzx/wzy* genes, meaning very likely that most *E. cloacae* strains utilize the Wzx/Wzy-dependent pathway for O-antigen translocation and polymerization. The only exception is O23, which possess *wzm*/*wzt* genes instead of *wzx*/*wzy* genes, suggesting that the O23-antigen is synthesized via the ABC transporter (Wzm/Wzt)-dependent pathway. An anomaly here is that only *wzy* gene is annotated in O13, and we propose that *wzx* gene of O13 must be located elsewhere in the chromosome. This atypical feature has been reported in other strains, such as *Klebsiella* K11 and K34 (Pan et al., 2015), and *Salmonella* serotypes A, B, and D1 (Wang et al., 2002).

### Development of a Multiplex PCR Assay

Compared with the nucleotide sugar precursor synthesis genes and glycosyltransferase genes, the O-antigen processing genes (*wzx*/*wzy* and *wzm*/*wzt*) are much more highly serotype-determinative (Li and Reeves, 2000; Ballmer et al., 2007). We constructed neighbor-joining phylogenetic trees for *wzx* and *wzy*, which showed high diversity levels among the different serotypes, except for O9/O10/O11, of which the O-AGCs shared 100% identity (Supplementary Figure S1). Therefore, *wzy* was selected as the target gene in terms of primer design for 25 of the 26 serotypes. Because *wzy* is lacking in the O-AGC of O23, *wzt* was selected instead. The 24 primer pairs were divided into three groups to generate target DNAs (Table 2).

**FIGURE 3:**
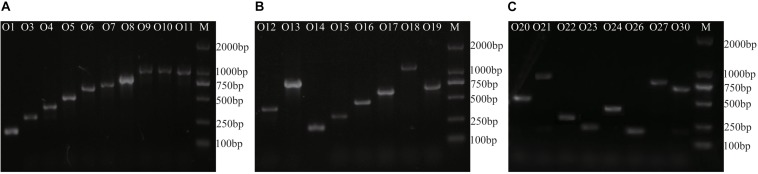
Agarose gel electrophoresis of representative Multiplex PCR products from 26 *E. cloacae* reference strains. M, DL2000 DNA molecular standard. **(A)** products of group 1, **(B)** products of group 2, and **(C)** products of group 3.

The multiplex PCR method was tested against each of the 26 O-standard *E. cloacae* reference strains and 16 strains of other species within the Enterobacteriaceae family (Table 1). In the presence of each target strain, only the corresponding sero-specific primer pair worked, and only one band of the expected size was generated (Figure 3). The amplicons ranged in size from 211 to 1,137 bp in length (Table 2). The representative *E. cloacae* strains belonging to other serotypes or other bacterial strains did not generate PCR products of the correct size. The results showed that all 24 primer pairs were specific and compatible in the multiplex PCR runs.

Furthermore, O3 from group 1, O17 from group 2, and O26 from group 3 were selected to determine the detection limit of our multiplex PCR assay. To determine the detection limit, serial 10-fold dilutions (10 ng to 0.1 pg) of genomic DNA from each strain were tested, which showed that the sensitivity of our assay was 0.1 ng for genomic DNA. To determine the sensitivity for pure cultures, the three serotypes were serially diluted 10-fold from 10^8^ down to 10^0^ colony forming units (CFUs) and used as templates for multiplex PCR. Our test demonstrated that positive signals could be generated for templates containing 10^3^ CFUs of pure culture.

A double-blinded test using 81 *E. cloacae* strains with unknown serotypes was performed to evaluate our multiplex PCR system. Among them, 73 were typeable, including 11 assigned to O1, 24 assigned to O3, 12 assigned to O9/10/11, and 10 assigned to O13, with the other serotypes each representing <10%. This result was confirmed to be correct by ABI3730 sequencing. The distribution of serotypes is generally consistent with Gaston’s study by using agglutination test against 300 clinical isolates (Gaston et al., 1983), with exception of O8, which accounted for >13% in his investigation.

### *In silico* Serotyping of Genomic Data for *E. cloacae* Strains

To evaluate our molecular serotyping scheme, we downloaded 431 *E. cloacae* genomes from GenBank and screened them using all sero-specific genes identified in our study. Among them, 304 could be assigned to certain serotypes, with O3 representing the predominant group (38%), followed by O8 (15%) and O13 (10%), and other serotypes assigned each <7%. The result of our *in silico* analysis is also in line with the allocation of serotypes studied by Gaston et al. (1983).

Among the remaining 127 genomes, the O-AGC was either not found or was too fragmented for 15 genomes, thus, these genomes were excluded from further analysis. The genetic region between *galF* and *gnd* in 112 strains was then extracted and analyzed, and 55 novel putative O-AGCs (temp 1–55) were obtained (Supplementary Table S2 and Supplementary Figure S2).

## Discussion

**FIGURE 4:**
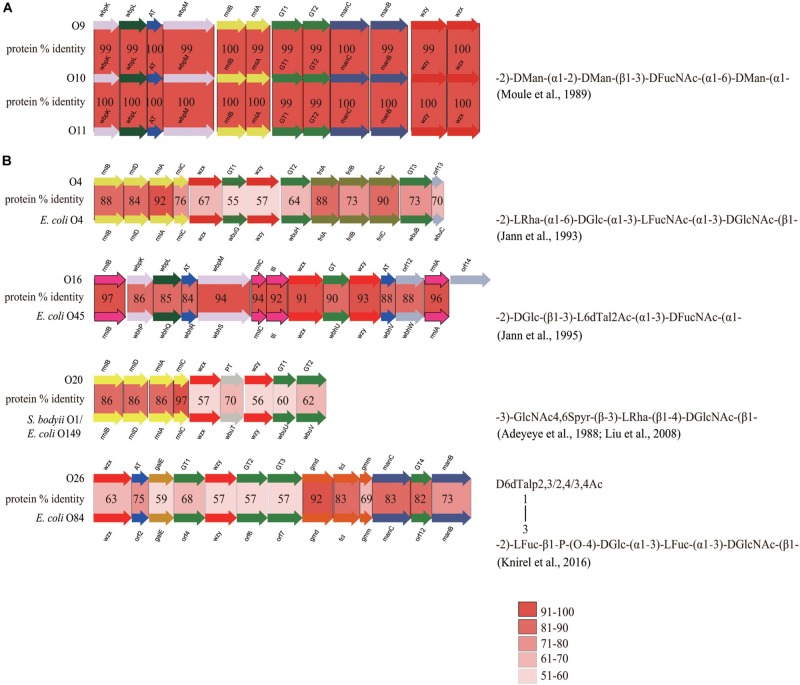
Comparison of closely related O-AGCs within *E. cloacae* strains and between *E. cloacae* and *E. coli* strains. **(A)** Comparison of O-AGCs of *E. cloacae* O9/O10/O11, with the O-antigen structure of O10 being shown. **(B)** Comparison of O-AGCs of *E. cloacae* O4 and *E. coli* O4, O16 and *E. coli* O45, O20 and *E. coli* O149/*S. bodyii* type 1, and O26 and *E. coli* O84, with the O-antigen structures of the latter in each group being shown.

At present, the O-antigen structure has only been elucidated for one reference strain (NCTC 11579, serotype O10), by Wilkinson’s group (Moule et al., 1989). The O-AGC of O10 in this study showed a perfect correlation with the structure (Figure 4A). In general, ManB and ManC, combined with ManA, are responsible for the formation of GDP-D-Man, the nucleotide sugar precursor of D-Man, whereas *manA* is always located outside of the O-AGC (Samuel and Reeves, 2003). WbpM and WbpK are responsible for the formation of UDP-D-FucNAc, the nucleotide sugar precursor of D-FucNAc (King et al., 2009). The products of two glycosyl transferase genes are proposed for the synthesis of two D-Man-(α1→2)-D-Man linkages and one D-Man-(β1→3)-D-FucNAc linkage, however, the exact functions of each could not be inferred. The presence of *wzx* and *wzy* genes probably means that the O-antigen of O10 is synthesized by the Wzx/Wzy-dependent pathway. We noticed that there is a D-Glc side branch attached to the β D-Man residue of the backbone of O10 antigen. This is commonly mediated by the Gtr process, which is involved by three enzymes, GtrA, GtrB, and GtrC, with all genes (*gtrA/B/C*) always being clustered in prophage genomes. GtrA and GtrB are highly conserved among different serotypes, and GtrC is unique to each serotype and is therefore the sero-specific glucosyl transferase (Allison and Verma, 2000; Wang et al., 2007). By screening the genome sequence of O10, we observed *gtrA* and *gtrB* homologs, but *gtrC* could not be annotated due to its low identity shared with the analogs. However, we consider that the gene just downstream of *gtrB* is most likely a *gtrC* gene unique to *E. cloacae* O10, as the protein encoded by it possesses 12 potential transmembrane domains, as predicted using TMHMM v2.0, being consistent with the topology of GtrC of *Shigella flexneri* (Korres and Verma, 2004). We also characterized the *gtr* gene set in O9 and O11, respectively. Pairwise comparison showed that the % identity level of GtrA among the three serotypes is 86–100, and GtrB 93–96, however, the % identity level of GtrC, the serotype determinant, ranges only from 30 to 34. In addition, O9, O10, and O11 possess almost identical O-AGCs with > 99% overall identity (Figure 4A), suggesting that the O-AGCs of these serotypes may be recently transferred from one isolate to the others and that their O-antigens very likely contain identical backbones. Although O9/O10/O11 appeared to represent an antigenic group, the distinct numbers for them were still be retained as high-titer-specific sera could be prepared by absorption (Gaston et al., 1983). Therefore, the minor antigenic difference among these serotypes must be accounted for by the variations in the side branches or modifications encoded by genes located elsewhere in the chromosome. On the other hand, the possibility could not be entirely excluded that each of the three serotypes possesses unique O-antigen structure, since a few gene-product pairs share 99% identity level which may influence the activity of them due to non-synonymous mutations, as the case in *E. coli* O9/O9a (Kido and Kobayashi, 2000).

In addition, we also observed a few strains whose O-AGCs are closely related to those of some serotypes of *E. coli*: they are O4 and *E. coli* O4 (Jann et al., 1993), O16 and *E. coli* O45 (Jann et al., 1995), O20 and *E. coli* O149/*S. bodyii* type 1 (Adeyeye et al., 1988; Liu et al., 2008), and O26 and *E. coli* O84 (Knirel et al., 2016) (Figure 4B). Overall, strains in each group share almost identical gene order, and significant protein identity level (55–97%). In these pairs of O-AGCs in *E. cloacae* and *E. coli* excluding the O16/*E. coli* O45 group, the average level of protein identity encoded by nucleotide sugar precursor synthesis genes, glycosyltransferase genes, and O-unit processing genes, is 82, 64, and 59%, respectively. We assume that each pair has evolved from a gene cluster located in a common ancestor, but that the three classes of genes underwent different selective pressures after divergence, as is the case in some *Salmonella* O-antigens (Liu et al., 2014). For the O16/*E. coli* O45 group, the overall identity of each pair of gene products is similar (84–97%), and we suppose that the O-AGCs of them probably also originated from a common ancestor recently and still underwent rapidly-evolving events. Elucidation of the O-antigen structures of more *E. cloacae* serotypes will undoubtedly enhance our understanding of the evolution of O-AGCs of this bacterium, as well as the genetic relatedness of intra- and inter-species.

Efforts have been made to develop a method for timely clinical diagnosis, epidemiological surveillance, and outbreak detection for *E. cloacae*, including biotyping, phage typing, and ribotyping (Gaston, 1987; Garaizar et al., 1991; Weischer et al., 1993). However, these assays are not highly discriminatory and reproducible methods for *E. cloacae* typing. Subsequently, a multilocus sequence-typing scheme was presented (Miyoshi-Akiyama et al., 2013) and employed to characterize *E. cloacae* isolates (Viau et al., 2017; Wang et al., 2017; Miao et al., 2019). More recently, WGS-based methods were presented, showing enhanced discriminative power and shedding new insights into the phylogeny and resistance mechanisms of *E. cloacae* (Chavda et al., 2016; Beyrouthy et al., 2018); however, the WGS data analysis has not been fully standardized.

Conventional serotyping by using agglutination test is always delicate, laborious, time-consuming, and expensive. For decades, several molecular assays targeting sero-specific genes and showing fast, reliable, and cost-effective detection were developed for bacterial serotyping (Azzari et al., 2010; Lin et al., 2011; Bai et al., 2015). Compared to normal PCR and Taqman probe-based real-time PCR methods that amplify individual or only at most four target gene(s), the multiplex PCR assay could simultaneously detect multiple targets in a single reaction, and generate the same accuracy while saving time and effort. Because of these advantages, multiplex PCR has been applied widely for the detection of many bacterial strains (van der Veer et al., 2018; Kwack et al., 2020; Collins et al., 2020). In the past few years, several WGS-based *in silico* serotyping approaches have been presented and showed better resolution compared to conventional methods, and have been utilized for epidemiological investigation and tracing (Thrane et al., 2016; Ibrahim and Morin, 2018; Wu et al., 2019). However, all of those studies are based on a key prerequisite that is the full and deep understanding of the O-antigen and the genetic basis for its diversity/variation. To date, the O-antigens and O-AGCs (or the capsular antigen and its genetic determinant) of several pathogenic species, especially in the Enterobacteriaceae family, including *Escherichia coli* (Iguchi et al., 2015), *Shigella* (Liu et al., 2008), *Salmonella* (Liu et al., 2014), *Klebsiella* (Pan et al., 2015), and *Yersinia pseudotuberculosis* (Kenyon et al., 2017) have been characterized in depth.

Although more O-antigen structures need to be elucidated to support our study, the work here, for the first time, presented the genetic basis regarding the O-antigen diversity and variation of *E. cloacae*, which also may partially help in understanding the evolution of this important pathogen. It should be noted, however, that using a conventional agglutination test or our multiplex PCR assay targeting only the present serotype groups, 10–23% isolates could not be assigned to certain serotypes, meaning that other novel serotypes are still evolving and remain to be discovered. Indeed, a large number of putative novel serotypes were characterized by screening the *E. cloacae* genomes deposited in GenBank. The antigenic scheme for *E. cloacae* has not been updated since the 1990s; therefore, our current findings have expanded the existing serotyping system for *E. cloacae*, which is significant for detection and epidemiological surveillance purposes for this important pathogen.

## Data Availability Statement

The datasets generated for this study can be found in the GenBank database under accession numbers MK595714 to MK595739.

## Author Contributions

XG and TH conceived the project and prepared the manuscript. YL and XW prepared the strain samples, preformed genome sequencing and bioinformatic analyses. JH conducted the *in silico* serotyping program and analyses. CX developed the multiplex PCR assay and performed the double-blinded test. All authors read and approved the final manuscript.

## Conflict of Interest

The authors declare that the research was conducted in the absence of any commercial or financial relationships that could be construed as a potential conflict of interest.

## Footnotes

1 https://www.phe-culturecollections.org.uk/products/bacteria/detail.jsp?refId=NCTC+11932&collection=nctc and https://www.phe-culturecollections.org.uk/products/bacteria/detail.jsp?refId=NCTC+11933&collection=nctc

2 www.ncbi.nlm.nih.gov/genbank

3 pfam.sanger.ac.uk

4 http://www.cbs.dtu.dk/services/TMHMM-2.0/
