# Supplementary material for: Genetic Characterization of the O-Antigen and Development of a Molecular Serotyping Scheme for Enterobacter cloacae
Source: Front Microbiol. 2020 Apr 28;11:727. doi: 10.3389/fmicb.2020.00727 (PMC7198725; doi:10.3389/fmicb.2020.00727)
Supplement: Supplementary file 1 [file Data_Sheet_1.doc]

Supplementary Data 1. The python script used for prediction of E. cloacae serotypes using whole genome data.

#!/usr/bin/python

# coding=utf-8

from Bio import SeqIO

import sys

if len(sys.argv)<3:

print "python cover -i_fasta blastn length_percent similartity "

exit()

d = dict((i.id,len(i.seq))for i in SeqIO.parse(open(sys.argv[1]),'fasta'))

fb = open(sys.argv[2])

for i in fb:

j = i.strip().split('\t')

if d.has_key(j[0]):

result = float(j[3])/int(d[j[0]])*100

if result >= int(sys.argv[3]):

if float(j[2]) >= int(sys.argv[4]):

print j[0],'\t',j[1],'\t',j[2],'\t',j[3],'\t',d[j[0]],'\t',result

fb.close()
