# Supplementary material for: Genetic Characterization of the O-Antigen and Development of a Molecular Serotyping Scheme for Enterobacter cloacae
Source: Front Microbiol. 2020 Apr 28;11:727. doi: 10.3389/fmicb.2020.00727 (PMC7198725; doi:10.3389/fmicb.2020.00727)
Supplement: Supplementary file 2 [file Image_1.pdf]

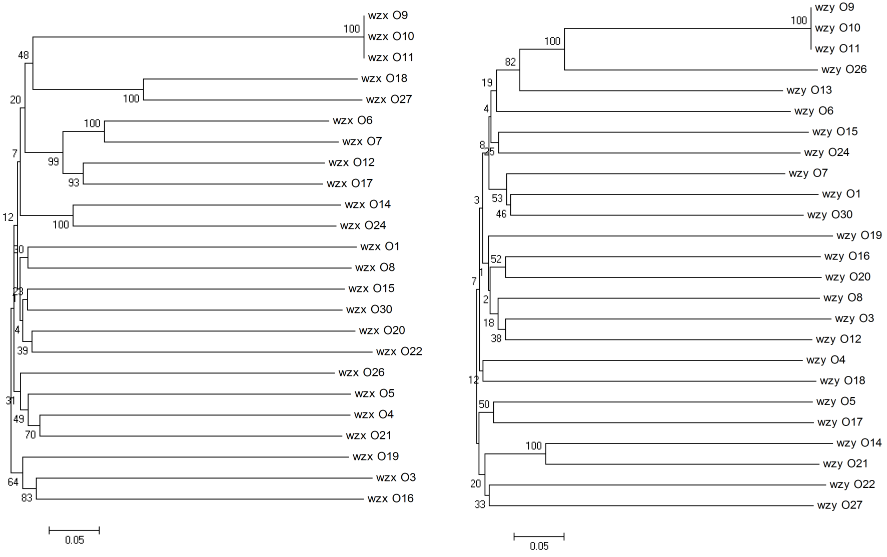

Supplementary figure 1. Neighbor-Joining phylogenetic tree for *wzx* (left) and *wzy* (right), respectively, using MEGA 4 with 1000 bootstraps.
