# Supplementary material for: Genetic Characterization of the O-Antigen and Development of a Molecular Serotyping Scheme for Enterobacter cloacae
Source: Front Microbiol. 2020 Apr 28;11:727. doi: 10.3389/fmicb.2020.00727 (PMC7198725; doi:10.3389/fmicb.2020.00727)
Supplement: Supplementary file 3 [file Image_2.pdf]

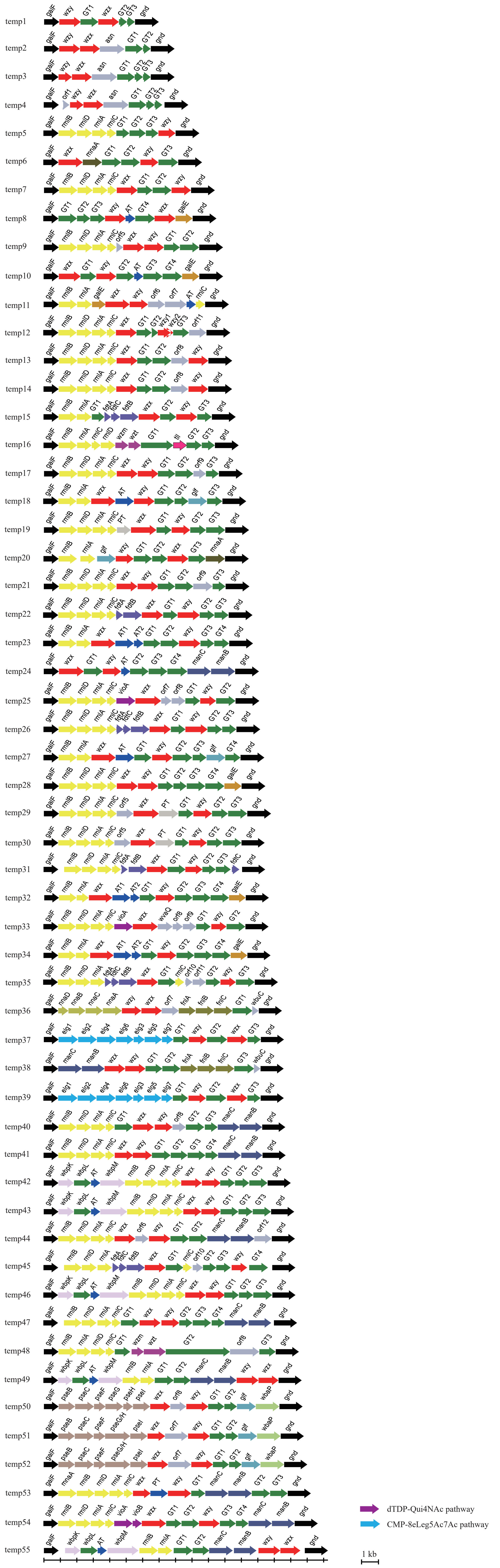

Supplementary figure 2. Schematic diagram of the putative O-AGCs characterized from the *E. cloacae* genomes from Genbank. Genes are represented by arrows and colored according to the gene key of Figure 1.
