# Supplementary material for: Genetic Characterization of the O-Antigen and Development of a Molecular Serotyping Scheme for Enterobacter cloacae
Source: Front Microbiol. 2020 Apr 28;11:727. doi: 10.3389/fmicb.2020.00727 (PMC7198725; doi:10.3389/fmicb.2020.00727)
Supplement: Supplementary file 4 [file Table_1.doc]

Supplementary Table 1. Characteristics of the ORFs in 26 *E. cloacae* O-antigen gene clusters

| *E. cloacae* O1 | | | | | | | |
| --- | --- | --- | --- | --- | --- | --- | --- |
| Orf no. | Gene name | Position of gene | G+C content(%) | Conserved domain(s) | Similar protein(s), strain(s) (Genbank accession No.) | %Identical/%Similar (total No. of aa) | Putative function of protein |
| 1 | *rmlB* | 1256..2341 | 51.47 | GDP_Man_Dehyd; PF16363.1; E value=5.1e-98 | dTDP-glucose 4,6-dehydratase [Enterobacter cloacae] (WP_072139452.1) | 99/99(361) | dTDP-glucose 4,6-dehydratase |
| 2 | *rmlD* | 2341..3240 | 51.89 | RmlD_sub_bind; PF04321.13; E value=3.4e-107 | dTDP-4-dehydrorhamnose reductase [Enterobacter cloacae] (WP_063148526.1) | 99/99(299) | dTDP-4-dehydrorhamnose reductase |
| 3 | *rmlA* | 3292..4170 | 49.26 | NTP_transferase; PF00483.19; E value=8.3e-74 | glucose-1-phosphate thymidylyltransferase RfbA [Enterobacter cloacae](WP_047955853.1) | 98/98(292) | Glucose-1-phosphate thymidylyltransferase |
| 4 | *rmlC* | 4174..4722 | 38.62 | dTDP_sugar_isom; PF00908.13; E value=5.3e-80 | dTDP-4-dehydrorhamnose 3,5-epimerase [Escherichia coli] (WP_096933633.1) | 88/95(182) | dTDP-4-dehydrorhamnose 3,5-epimerase |
| 5 | *wzx* | 4865..6010 | 34.12 | Polysacc_synt; PF01943.13; E value=7.8e-09 | Wzx [Shigella boydii] (ACD37025.1) | 46/66(381) | flippase |
| 6 | *wzy* | 5979..7148 | 32.74 | O-antigen_lig; PF13425.2; E value=2.3e-05 | Wzy [Shigella boydii] (ACD37026.1) | 48/67(389) | O-antigen polymerase |
| 7 | *GT1* | 7141..8283 | 31.58 | Glycos_transf_1; PF00534.16; E value=1.4e-17 | glycosyltransferase family 1 protein [Chroococcidiopsis thermalis] (WP_015152917.1) | 28/48(380) | UDP-D-galactose:(glucosyl)lipopolysaccharide-1, 6-D-galactosyltransferase |
| 8 | *GT2* | 8287..9318 | 32.07 | Glycos_transf_1; PF00534.16; E value=5.1e-21 | glycosyltransferase family 1 protein [Escherichia coli] (WP_077783474.1) | 86/94(343) | Glycosyl transferases group 1 |
| 9 | *GT3* | 9315..10397 | 33.52 | Glycos_transf_1; PF00534.16; E value=2e-23 | group 1 glycosyl transferase [Klebsiella pneumoniae] (CZQ24902.1) | 30/50(360) | GDP-mannose-dependent alpha-mannosyltransferase |
| 10 | *GT4* | 10406..11176 | 31.0 | Glycos_transf_2; PF00535.22; E value=0.00017 | glycosyl transferase family 2 [Escherichia coli] (KHH21241.1) | 82/90(256) | Rhamnosyltransferase WbbL |
| 11 | *manC* | 11173..12558 | 37.73 | NTP_transferase; PF00483.19; E value=3.8e-68 | mannose-1-phosphate guanylyltransferase [Escherichia coli] (BAU71577.1) | 88/94(461) | Mannose-1-phosphate guanylyltransferase |
| 12 | *manB* | 12566..13936 | 59.01 | PGM_PMM_I; PF02878.12; E value=1.4e-35 | phosphomannomutase CpsG [Enterobacter cloacae] (WP_063932718.1) | 99/100(456) | Phosphomannomutase/phosphoglucomutase |

| *E. cloacae* O3 | | | | | | | |
| --- | --- | --- | --- | --- | --- | --- | --- |
| Orf no. | Gene name | Position of gene | G+C content(%) | Conserved domain(s) | Similar protein(s), strain(s) (Genbank accession No.) | %Identical/%Similar (total No. of aa) | Putative function of protein |
| 1 | *manC* | 1225..2622 | 48.57 | NTP_transferase; PF00483.19; E value=8.2e-70 | mannose-1-phosphate guanylyltransferase/mannose-6-phosphate isomerase [Enterobacter hormaechei] (WP_049108488.1) | 99/99(465) | Mannose-1-phosphate guanylyltransferase |
| 2 | *manB* | 2622..4004 | 47.94 | PGM_PMM_I; PF02878.12; E value=1.3e-35 | phosphomannomutase/phosphoglucomutase [Enterobacter hormaechei] (WP_058658063.1) | 99/99 (460) | Phosphomannomutase/phosphoglucomutase |
| 3 | *wzy* | 4039..5358 | 42.88 | O-antigen_lig; PF13425.2; E value=3.1e-17 | O-antigen polymerase [Klebsiella variicola] (CTQ06010.1) | 49/70(439) | O-Antigen polymerase |
| 4 | *GT1* | 5355..6491 | 42.57 | Glycos_transf_1; PF00534.16; E value=6.1e-44 | glycosyl transferase family 1 [Enterobacter cloacae] (WP_032665784.1) | 80/89(378) | GDP-mannose-dependent alpha-(1-6)-phosphatidylinositol monomannoside mannosyltransferase |
| 5 | *GT2* | 6484..7650 | 36.08 | Glycos_transf_1; PF00534.16; E value=3.3e-29 | glycosyltransferase family 1 protein [Enterobacter cloacae] (WP_088569474.1) | 71/85(388) | Putative glycosyltransferase EpsF |
| 6 | *wzx* | 7647..9140 | 38.62 | MVIN; PF03023.10; E value=2.2e-34 | flippase [Klebsiella pneumoniae] (SCA95783.1) | 55/74(497) | flippase |
| 7 | *GT3* | 9150..10349 | 45.58 | Glyco_trans_1_4; PF13692.2; E value=4e-17 | glycosyltransferase family 1 protein [Enterobacter cancerogenus] (WP_088208143.1) | 85/92(399) | Putative teichuronic acid biosynthesis glycosyltransferase TuaH |
| 8 | *GT4* | 10327..11508 | 43.99 | Glycos_transf_1; PF00534.16; E value=4.1e-25 | glycosyltransferase family 4 protein [Kosakonia sacchari] (WP_074778183.1) | 86/95(393) | GDP-mannose-dependent alpha-(1-6)-phosphatidylinositol monomannoside mannosyltransferase |
| 9 | *wbaP* | 11552..12988 | 40.57 | Bac_transf; PF02397.12; E value=1.2e-56 | undecaprenyl-phosphate galactose phosphotransferase WbaP, partial [Enterobacter cloacae] (WP_047930446.1) | 99/99(478) | undecaprenyl-phosphate galactose phosphotransferase |
| 10 | *GT5* | 13028..14059 | 36.34 | Glyco_trans_1_4; PF13692.2; E value=7.5e-07 | glycosyltransferase family 1 protein [Kosakonia sacchari] (WP_074778179.1) | 59/74(343) | glycosyltransferase |
| 11 | *rmlB* | 14139..15203 | 42.72 | GDP_Man_Dehyd; PF16363.1; E value=6.3e-93 | dTDP-glucose 4,6-dehydratase [Enterobacter cloacae] (WP_063139132.1) | 99/100(354) | dTDP-glucose 4,6-dehydratas |
| 12 | *rmlA* | 15219..16085 | 44.06 | NTP_transferase; PF00483.19; E value=1.1e-69 | glucose-1-phosphate thymidylyltransferase [Enterobacter cloacae] (WP_100148664.1) | 99/100(288) | Glucose-1-phosphate thymidylyltransferase |
| 13 | *rmlD* | 16092..16988 | 45.71 | RmlD_sub_bind; PF04321.13; E value=8.8e-101 | dTDP-4-dehydrorhamnose reductase [Enterobacter cloacae] (CZV32473.1) | 99/100(298) | dTDP-4-dehydrorhamnose reductase |
| 14 | *rmlC* | 16999..17547 | 41.17 | dTDP_sugar_isom; PF00908.13; E value=3.8e-72 | dTDP-4-dehydrorhamnose 3,5-epimerase [Enterobacter cloacae] (WP_058660576.1) | 99/100 (182) | dTDP-4-dehydrorhamnose 3,5-epimerase |

| *E. cloacae* O4 | | | | | | | |
| --- | --- | --- | --- | --- | --- | --- | --- |
| Orf no. | Gene name | Position of gene | G+C content(%) | Conserved domain(s) | Similar protein(s), strain(s) (Genbank accession No.) | %Identical/%Similar (total No. of aa) | Putative function of protein |
| 1 | *rmlB* | 1256..2341 | 51.47 | NTP_transferase; PF00483.19; E value=4.2e-23 | dTDP-glucose 4,6-dehydratase [Enterobacter hormaechei] (WP_096946996.1) | 99/100(361) | dTDP-glucose 4,6-dehydratase |
| 2 | *rmlD* | 2341..3240 | 51.67 | GDP_Man_Dehyd; PF16363.1; E value=3.5e-98 | dTDP-4-dehydrorhamnose reductase [Enterobacter cloacae] (WP_063855582.1) | 99/98(299) | dTDP-4-dehydrorhamnose reductase |
| 3 | *rmlA* | 3293..4171 | 50.06 | RmlD_sub_bind; PF04321.13; E value=1.8e-108 | glucose-1-phosphate thymidylyltransferase RfbA [Enterobacter cloacae] (WP_063928943.1) | 97/99(292) | Glucose-1-phosphate thymidylyltransferase |
| 4 | *rmlC* | 4173..4721 | 36.61 | NTP_transferase; PF00483.19; E value=1e-73 | dTDP-4-dehydrorhamnose 3,5-epimerase [Escherichia coli] (WP_096976440.1) | 70/81(182) | dTDP-4-dehydrorhamnose 3,5-epimerase |
| 5 | *wzx* | 4734..5972 | 34.38 | dTDP_sugar_isom; PF00908.13; E value=3.3e-74 | flippase [Escherichia coli] (WP_086167133.1) | 67/83(412) | flippase |
| 6 | *GT1* | 5969..6868 | 32.89 | Polysacc_synt; PF01943.13; E value=3.6e-30 | glycosyltransferase family 2 protein [Leclercia adecarboxylata] (WP_048030834.1) | 75/85(299) | UDP-Glc:alpha-D-GlcNAc-diphosphoundecaprenol beta-1,3-glucosyltransferase |
| 7 | *wzy* | 6865..8052 | 29.29 | Glycos_transf_2; PF00535.22; E value=5.1e-25 | O antigen polymerase [Escherichia coli J96] (ELL42069.1) | 56/76(395) | polymerase |
| 8 | *GT2* | 8122..9225 | 32.7 | EpsG; PF14897.2; E value=6.5e-38 | glycosyltransferase family 1 protein [Escherichia coli] (WP_000459968.1) | 64/80(367) | Glycosyltransferase Gtf1 |
| 9 | *fnlA* | 9235..10269 | 41.26 | Glycos_transf_1; PF00534.16; E value=2.2e-21 | UDP-N-acetylglucosamine 4,6-dehydratase/5-epimerase [Cronobacter universalis] (WP_007699418.1) | 92/95(344) | UDP-N-acetylglucosamine 4,6-dehydratase/5-epimerase |
| 10 | *fnlB* | 10271..11377 | 43.36 | Polysacc_synt_2; PF02719.11; E value=7e-105 | UDP-2-acetamido-2,6-dideoxy-beta-L-talose 4-dehydrogenase [Escherichia coli] (WP_000697810.1) | 90/95(368) | UDP-2-acetamido-2,6-beta-L-arabino-hexul-4-ose reductase |
| 11 | *fnlC* | 11374..12504 | 44.39 | Epimerase; PF01370.17; E value=1.6e-18 | FnlC [Escherichia coli] (AAV74546.1) | 95/97(376) | UDP-2,3-diacetamido-2,3-dideoxy-D-glucuronate 2-epimerase |
| 12 | *GT3* | 12504..13715 | 44.06 | Epimerase_2; PF02350.15; E value=1.8e-91 | glycosyltransferase WbuB [Enterobacter cancerogenus] (WP_101737345.1) | 91/93(403) | putative glycosyl transferase |
| 13 | *orf13* | 13702..14100 | 42.36 | Glyco_trans_1_4; PF13692.2; E value=4.4e-10 | cupin fold metalloprotein, WbuC family [Escherichia coli] (WP_089529458.1) | 80/94(132) | cupin fold metallo protein, WbuC |

| *E. cloacae* O5 | | | | | | | |
| --- | --- | --- | --- | --- | --- | --- | --- |
| Orf no. | Gene name | Position of gene | G+C content(%) | Conserved domain(s) | Similar protein(s), strain(s) (Genbank accession No.) | %Identical/%Similar (total No. of aa) | Putative function of protein |
| 1 | *nnaD* | 1226..1858 | 39.81 | Hexapep; PF00132.20; E value=5.6e-06 | sialic acid synthase [Escherichia coli] (WP_000810675.1) | 74/85(210) | Putative acetyltransferase |
| 2 | *nnaB* | 1851..2891 | 36.7 | NeuB; PF03102.10; E value=6.3e-85 | NeuNAc condensing enzyme [Escherichia coli] (AAX58758.1) | 85/93(346) | N,N'-diacetyllegionaminic acid synthase |
| 3 | *nnaC* | 2898..4160 | 33.81 | CTP_transf_3; PF02348.15; E value=2.9e-33 | N-acylneuraminate cytidylyltransferase [Salmonella enterica] (WP_000053698.1) | 63/78(420) | N-acylneuraminate cytidylyltransferase |
| 4 | *nnaA* | 4157..5311 | 36.1 | Epimerase_2; PF02350.15; E value=1.2e-72 | UDP-N-acetylglucosamine 2-epimerase (hydrolyzing) [Escherichia coli] (WP_047084279.1) | 72/86(384) | UDP-N-acetylglucosamine 2-epimerase |
| 5 | *wzx* | 5339..6595 | 36.83 | Polysacc_synt; PF01943.13; E value=2.6e-20 | Wzx [Cronobacter turicensis] (AFO84313.1) | 48/72(418) | flippase |
| 6 | *wzy* | 6688..8025 | 34.16 | Hexapep; PF00132.20; E value=5.6e-06 | O antigen polymerase Wzy [Escherichia coli] (ADN43883.1) | 26/45(445) | O antigen polymerase |
| 7 | *GT1* | 8025..8987 | 28.45 |  | lipooligosaccharide sialyltransferase [Streptococcus suis] (WP_044767703.1) | 33/62(320) | lipooligosaccharide sialyltransferase |
| 8 | *GT2* | 9034..9759 | 37.19 | Gly_transf_sug; PF04488.11; E value=4.1e-17 | glycosyl transferase [Bacteroides fragilis] (WP_005802414.1) | 59/72(241) | glycosyl transferase |
| 9 | *mnaA* | 9759..10856 | 40.71 | Epimerase_2; PF02350.15; E value=9.1e-124 | UDP-N-acetylglucosamine 2-epimerase (non-hydrolyzing) [Clostridium aceticum] (WP_044824653.1) | 84/92(365) | UDP-N-acetylglucosamine 2-epimerase |
| 10 | *GT3* | 10938..11744 | 34.94 | Glycos_transf_2; PF00535.22; E value=1.3e-19 | glycosyl transferase 2 family protein [Escherichia coli] (WP_001402316.1) | 63/81(268) | UDP-Gal:alpha-D-GlcNAc-diphosphoundecaprenol beta-1,3-galactosyltransferase |

| *E. cloacae* O6 | | | | | | | |
| --- | --- | --- | --- | --- | --- | --- | --- |
| Orf no. | Gene name | Position of gene | G+C content(%) | Conserved domain(s) | Similar protein(s), strain(s) (Genbank accession No.) | %Identical/%Similar (total No. of aa) | Putative function of protein |
| 1 | *wzx* | 1032..2279 | 31.25 | Polysacc_synt; PF01943.13; E value=9.8e-37 | O-antigen flippase [Escherichia coli] (BAQ00595.1) | 68/84(415) | flippase |
| 2 | *GT1* | 2269..3198 | 29.89 | Glycos_transf_2; PF00535.22; E value=2.8e-35 | glycosyltransferase family 2 protein [Escherichia coli] (WP_001597953.1) | 60/73(309) | Putative glycosyltransferase |
| 3 | *wzy* | 3198..4448 | 28.46 | O-antigen_lig; PF13425.2; E value=3.2e-07 | O8 family O-antigen polymerase [Escherichia coli] (WP_001597952.1) | 64/80(416) | O-antigen polymerase |
| 4 | *AT* | 4438..4884 | 37.36 | Hexapep_2; PF14602.2; E value=6.9e-10 | acyltransferase [Escherichia coli] (WP_001597951.1) | 74/84(148) | putative acyl transferase |
| 5 | *GT2* | 4887..6041 | 31.34 | Glycos_transf_1; PF00534.16; E value=1.1e-21 | putative glycosyltransferase [Escherichia coli] (BAQ00598.1) | 58/72(384) | glycosyltransferase |
| 6 | *GT3* | 6067..6819 | 32.14 | Glycos_transf_2; PF00535.22; E value=2.1e-35 | glycosyltransferase family 2 protein [Escherichia coli] (WP_001597949.1) | 66/79(250) | glycosyltransferase |

| *E. cloacae* O7 | | | | | | | |
| --- | --- | --- | --- | --- | --- | --- | --- |
| Orf no. | Gene name | Position of gene | G+C content(%) | Conserved domain(s) | Similar protein(s), strain(s) (Genbank accession No.) | %Identical/%Similar (total No. of aa) | Putative function of protein |
| 1 | *wzx* | 1374..2630 | 33.73 | Polysacc_synt; PF01943.13; E value=4.6e-43 | O28ab family O-antigen flippase [Escherichia coli] (WP_040090292.1) | 41/63(418) | flippase |
| 2 | *GT1* | 2623..3603 | 29.26 | Glycos_transf_2; PF00535.22; E value=4.7e-32 | putative glycosyltransferase EpsJ [Hafnia alvei] (ANF29953.1) | 52/69(326) | putative glycosyltransferase |
| 3 | *GT2* | 3649..4740 | 31.23 | Glycos_transf_1; PF00534.16; E value=2.2e-40 | glycosyltransferase family 4 protein [Enterobacter hormaechei] (WP_063443906.1) | 84/92(363) | GalNAc-alpha-(1->4)-GalNAc-alpha-(1->3)- diNAcBac-PP-undecaprenol alpha-1,4-N-acetyl-D-galactosaminyltransferase |
| 4 | *wzy* | 4740..5888 | 27.94 | EpsG; PF14897.2; E value=1e-29 | Wzy [Acinetobacter baumannii] (AHB32215.1) | 50/75(382) | O antigen polymerase |
| 5 | *GT3* | 5892..6995 | 29.89 | Glycos_transf_1; PF00534.16; E value=4.1e-21 | glycosyltransferase family 1 protein [Escherichia coli] (WP_039022705.1) | 60/81(367) | N, N'-diacetylbacillosaminyl-diphospho-undecaprenol alpha-1,3-N-acetylgalactosaminyltransferase |
| 6 | *GT4* | 7029..8084 | 30.3 | Glycos_transf_1; PF00534.16; E value=7.7e-28 | glycosyltransferase family 1 protein [Colwellia sediminilitoris] (WP_085329333.1) | 38/58(351) | UDP-D-galactose:(glucosyl)lipopolysaccharide-1, 6-D-galactosyltransferase |
| 7 | *manC* | 8086..9507 | 40.37 | MannoseP_isomer; PF01050.14; E value=2.7e-74 | mannose-1-phosphate guanylyltransferase/mannose-6-phosphate isomerase [Enterobacter hormaechei] (WP_059444797.1) | 91/96(473) | Mannose-1-phosphate guanylyltransferase |
| 8 | *manB* | 9597..10988 | 59.41 | PGM_PMM_I; PF02878.12; E value=1.3e-35 | phosphomannomutase [Enterobacter cloacae] (SAD05096.1) | 97/98(463) | Phosphomannomutase/phosphoglucomutase |

| *E. cloacae* O8 | | | | | | | |
| --- | --- | --- | --- | --- | --- | --- | --- |
| Orf no. | Gene name | Position of gene | G+C content(%) | Conserved domain(s) | Similar protein(s), strain(s) (Genbank accession No.) | %Identical/%Similar (total No. of aa) | Putative function of protein |
| 1 | *GT1* | 1182..2279 | 34.52 | Glycos_transf_1; PF00534.16; E value=5e-37 | glycosyltransferase family 1 protein [Escherichia coli] (WP_050864011.1) | 59/74(365) | O-antigen biosynthesis glycosyltransferase |
| 2 | *GT2* | 2276..3064 | 32.57 | Glycos_transf_2; PF00535.22; E value=1.6e-12 | glycosyl transferase family protein [Vibrio campbellii] (WP_010647951.1) | 38/58(262) | O-antigen biosynthesis glycosyltransferase |
| 3 | *GT3* | 3061..3945 | 32.2 | Glycos_transf_2; PF00535.22; E value=2.3e-20 | glycosyltransferase family 2 protein, partial [Escherichia coli] (WP_086258672.1) | 59/73(294) | glycosyltransferas |
| 4 | *wzy* | 3935..5170 | 32.04 | O-antigen_lig; PF13425.2; E value=1.9e-11 | O22 family O-antigen polymerase [Escherichia coli] (WP_097467265.1) | 53/68(411) | O-antigen polymerase |
| 5 | *AT* | 5142..5723 | 36.08 | Hexapep; PF00132.20; E value=7.5e-13 | galactoside O-acetyltransferase [Hafnia alvei] (ANF29886.1) | 63/78(193) | Galactoside O-acetyltransferase |
| 6 | *GT4* | 5720..6883 | 32.39 | Glycos_transf_1; PF00534.16; E value=9.7e-34 | glycosyltransferase family 4 protein [Escherichia coli] (WP_001533463.1) | 56/72(387) | glycosyltransferase family 4 |
| 7 | *wzx* | 6861..8093 | 32.85 | Polysacc_synt_3; PF13440.2; E value=4e-09 | O22 family O-antigen flippase [Escherichia coli] (WP_097727927.1) | 49/70(410) | flippase |
| 8 | *gne* | 8112..9125 | 36.88 | Epimerase; PF01370.17; E value=4.9e-51 | UDP-glucose 4-epimerase GalE [Escherichia coli] (WP_000697852.1) | 67/77(337) | UDP-glucose 4-epimerase |

| *E. cloacae* O9 | | | | | | | |
| --- | --- | --- | --- | --- | --- | --- | --- |
| Orf no. | Gene name | Position of gene | G+C content(%) | Conserved domain(s) | Similar protein(s), strain(s) (Genbank accession No.) | %Identical/%Similar (total No. of aa) | Putative function of protein |
| 1 | *wbpK* | 1287..2231 | 43.39 | Epimerase; PF01370.17; E value=4.6e-28 | NAD-dependent epimerase/dehydratase [Enterobacter sp. Ag1] (EJF32346.1) | 60/78(314) | NAD-dependent epimerase/dehydratase |
| 2 | *wbpL* | 2231..3274 | 46.55 | Glycos_transf_4; PF00953.17; E value=7.8e-29 | polyprenol phosphate:N-acetyl-hexosamine 1-phosphate transferase [Yersinia frederiksenii] (CNF58510.1) | 53/70(347) | polyprenol phosphate:N-acetyl-hexosamine 1-phosphate transferase |
| 3 | *AT* | 3267..3827 | 46.88 | Hexapep; PF00132.20; E value=4.9e-08 | sugar O-acyltransferase [Enterobacter cloacae complex sp. 35669] (WP_045260407.1) | 99/99(186) | UDP-N-acetylbacillosamine N-acetyltransferase |
| 4 | *wbpM* | 3866..5752 | 46.74 | Polysacc_synt_2; PF02719.11; E value=5.9e-127 | nucleoside-diphosphate sugar epimerase [Enterobacter cloacae complex sp. 35669] (KJN34375.1) | 98/98(628) | nucleoside-diphosphate sugarepimerase/dehydratase |
| 5 | *rmlB* | 5855..6937 | 43.49 | GDP_Man_Dehyd; PF16363.1; E value=4.4e-97 | dTDP-glucose 4,6-dehydratase [Enterobacter cloacae] (WP_048992939.1) | 92/95(360) | dTDP-glucose 4,6-dehydratase |
| 6 | *rmlA* | 6940..7806 | 37.83 | NTP_transferase; PF00483.19; E value=2.2e-68 | glucose-1-phosphate thymidylyltransferase [Enterobacter cloacae] (WP_063259246.1) | 99/99(288) | Glucose-1-phosphate thymidylyltransferase |
| 7 | *GT1* | 7888..9045 | 32.47 | Glyco_trans_1_4; PF13692.2; E value=1.9e-20 | glycosyl transferases group 1 [Escherichia coli] (AIG62739.1) | 59/76(385) | Glycosyl transferases group 1 |
| 8 | *GT2* | 9042..10061 | 32.45 | Glycos_transf_1; PF00534.16; E value=1.5e-08 | glycosyl transferases group 1 [Escherichia coli] (AIG62740.1) | 66/77(339) | Glycosyl transferases group 1 |
| 9 | *manC* | 10043..11479 | 42.38 | NTP_transferase; PF00483.19; E value=1.3e-75 | mannose-1-phosphate guanylyltransferase/mannose-6-phosphate isomerase [Enterobacter cloacae] (WP_048992937.1) | 95/97(478) | Mannose-1-phosphate guanylyltransferase |
| 10 | *manB* | 11549..12919 | 40.85 | PGM_PMM_I; PF02878.12; E value=8.1e-36 | phosphomannomutase [Enterobacter cloacae] (WP_048992936.1) | 94/96(456) | Phosphomannomutase/phosphoglucomutase |
| 11 | *wzy* | 13041..14369 | 30.32 |  | O-antigen polymerase [Salmonella enterica] (WP_023183905.1) | 55/74(442) | O-antigen polymerase |
| 12 | *wzx* | 14371..15588 | 31.61 | Polysacc_synt_C; PF14667.2; E value=6.5e-05 | O-antigen filppase [Salmonella enterica] (WP_023234587.1) | 53/70(405) | O-antigen filppase |

| *E. cloacae* O10 | | | | | | | |
| --- | --- | --- | --- | --- | --- | --- | --- |
| Orf no. | Gene name | Position of gene | G+C content(%) | Conserved domain(s) | Similar protein(s), strain(s) (Genbank accession No.) | %Identical/%Similar (total No. of aa) | Putative function of protein |
| 1 | *wbpK* | 1287..2231 | 43.39 | Epimerase; PF01370.17; E value=4.6e-28 | NAD-dependent epimerase/dehydratase [Enterobacter sp. Ag1] (EJF32346.1) | 60/78(314) | NAD-dependent epimerase/dehydratase |
| 2 | *wbpL* | 2231..3274 | 46.65 | Glycos_transf_4; PF00953.17; E value=7.2e-29 | polyprenol phosphate:N-acetyl-hexosamine 1-phosphate transferase [Yersinia frederiksenii] (CNF58510.1) | 53/70(347) | polyprenol phosphate:N-acetyl-hexosamine 1-phosphate transferase |
| 3 | *AT* | 3267..3827 | 46.88 | Hexapep; PF00132.20; E value=4.9e-08 | sugar O-acyltransferase [Enterobacter cloacae complex sp. 35669] (WP_045260407.1) | 99/99(186) | UDP-N-acetylbacillosamine N-acetyltransferase |
| 4 | *wbpM* | 3866..5752 | 46.48 | Polysacc_synt_2; PF02719.11; E value=4.4e-127 | nucleoside-diphosphate sugar epimerase [Enterobacter cloacae complex sp. 35669] (KJN34375.1) | 98/98(628) | nucleoside-diphosphate sugarepimerase/dehydratase |
| 5 | *rmlB* | 5855..6937 | 43.49 | GDP_Man_Dehyd; PF16363.1; E value=4.4e-97 | dTDP-glucose 4,6-dehydratase [Enterobacter cloacae] (WP_048992939.1) | 92/95(360) | dTDP-glucose 4,6-dehydratase |
| 6 | *rmlA* | 6940..7806 | 37.83 | NTP_transferase; PF00483.19; E value=2.2e-68 | glucose-1-phosphate thymidylyltransferase [Enterobacter cloacae] (WP_063259246.1) | 99/99(288) | Glucose-1-phosphate thymidylyltransferase |
| 7 | *GT1* | 7888..9045 | 32.47 | Glyco_trans_1_4; PF13692.2; E value=1.9e-20 | glycosyl transferases group 1 [Escherichia coli] (AIG62739.1) | 59/76(385) | Glycosyl transferases group 1 |
| 8 | *GT2* | 9042..10061 | 32.35 | Glycos_transf_1; PF00534.16; E value=1.4e-08 | glycosyl transferases group 1 [Escherichia coli] (AIG62740.1) | 66/77(339) | Glycosyl transferases group 1 |
| 9 | *manC* | 10043..11479 | 42.38 | NTP_transferase; PF00483.19; E value=1.3e-75 | mannose-1-phosphate guanylyltransferase/mannose-6-phosphate isomerase [Enterobacter cloacae] (WP_048992937.1) | 95/97(478) | Mannose-1-phosphate guanylyltransferase |
| 10 | *manB* | 11549..12919 | 40.85 | PGM_PMM_I; PF02878.12; E value=8e-36 | phosphomannomutase [Enterobacter cloacae] (WP_048992936.1) | 94/96(456) | Phosphomannomutase/phosphoglucomutase |
| 11 | *wzy* | 13041..14369 | 30.17 |  | O-antigen polymerase [Salmonella enterica] (WP_023183905.1) | 55/74(442) | O-antigen polymerase |
| 12 | *wzx* | 14371..15588 | 31.61 | Polysacc_synt_C; PF14667.2; E value=6.5e-05 | O-antigen flippase [Salmonella enterica] (WP_023234587.1) | 53/70 (405) | O-antigen flippase |

| *E. cloacae* O11 | | | | | | | |
| --- | --- | --- | --- | --- | --- | --- | --- |
| Orf no. | Gene name | Position of gene | G+C content(%) | Conserved domain(s) | Similar protein(s), strain(s) (Genbank accession No.) | %Identical/%Similar (total No. of aa) | Putative function of protein |
| 1 | *wbpK* | 1287..2231 | 43.39 | Epimerase; PF01370.17; E value=4.6e-28 | NAD-dependent epimerase/dehydratase [Enterobacter sp. Ag1] (EJF32346.1) | 60/78(314) | NAD-dependent epimerase/dehydratase |
| 2 | *wbpL* | 2231..3274 | 46.65 | Glycos_transf_4; PF00953.17; E value=7.2e-29 | polyprenol phosphate:N-acetyl-hexosamine 1-phosphate transferase [Yersinia frederiksenii] (CNF58510.1) | 53/70(347) | polyprenol phosphate:N-acetyl-hexosamine 1-phosphate transferase |
| 3 | *AT* | 3267..3827 | 46.88 | Hexapep; PF00132.20; E value=4.9e-08 | sugar O-acyltransferase [Enterobacter cloacae complex sp. 35669] (WP_045260407.1) | 99/99(186) | UDP-N-acetylbacillosamine N-acetyltransferase |
| 4 | *wbpM* | 3866..5752 | 46.48 | Polysacc_synt_2; PF02719.11; E value=4.4e-127 | nucleoside-diphosphate sugar epimerase [Enterobacter cloacae complex sp. 35669] (KJN34375.1) | 98/98(628) | nucleoside-diphosphate sugarepimerase/dehydratase |
| 5 | *rmlB* | 5855..6937 | 43.49 | GDP_Man_Dehyd; PF16363.1; E value=4.4e-97 | dTDP-glucose 4,6-dehydratase [Enterobacter cloacae] (WP_048992939.1) | 92/95(360) | dTDP-glucose 4,6-dehydratase |
| 6 | *rmlA* | 6940..7806 | 37.72 | NTP_transferase; PF00483.19; E value=3.1e-68 | glucose-1-phosphate thymidylyltransferase [Enterobacter cloacae] (WP_063259246.1) | 99/99(288) | Glucose-1-phosphate thymidylyltransferase |
| 7 | *GT1* | 7888..9045 | 32.73 | Glyco_trans_1_4; PF13692.2; E value=2e-20 | glycosyl transferases group 1 [Escherichia coli] (AIG62739.1) | 59/76(385) | Glycosyl transferases group 1 |
| 8 | *GT2* | 9042..10061 | 32.45 | Glycos_transf_1; PF00534.16; E value=1.4e-08 | glycosyl transferases group 1 [Escherichia coli] (AIG62740.1) | 66/77(339) | Glycosyl transferases group 1 |
| 9 | *manC* | 10043..11479 | 42.38 | NTP_transferase; PF00483.19; E value=1.3e-75 | mannose-1-phosphate guanylyltransferase/mannose-6-phosphate isomerase [Enterobacter cloacae] (WP_048992937.1) | 95/97(478) | Mannose-1-phosphate guanylyltransferase |
| 10 | *manB* | 11549..12919 | 40.85 | PGM_PMM_I; PF02878.12; E value=8e-36 | phosphomannomutase [Enterobacter cloacae] (WP_048992936.1) | 94/96(456) | Phosphomannomutase/phosphoglucomutase |
| 11 | *wzy* | 13041..14369 | 30.17 |  | O-antigen polymerase [Salmonella enterica] (WP_023183905.1) | 55/74(442) | O-antigen polymerase |
| 12 | *wzx* | 14371..15588 | 31.61 | Polysacc_synt_C; PF14667.2; E value=6.5e-05 | O-antigen flippase [Salmonella enterica] (WP_023234587.1) | 53/70 (405) | O-antigen flippase |

| *E. cloacae* O12 | | | | | | | |
| --- | --- | --- | --- | --- | --- | --- | --- |
| Orf no. | Gene name | Position of gene | G+C content(%) | Conserved domain(s) | Similar protein(s), strain(s) (Genbank accession No.) | %Identical/%Similar (total No. of aa) | Putative function of protein |
| 1 | *wbpK* | 1279..2223 | 45.5 | Epimerase; PF01370.17; E value=5.3e-28 | NAD-dependent epimerase/dehydratase [Enterobacter sp. Ag1] (EJF32346.1) | 60/78(314) | NAD-dependent epimerase/dehydratase |
| 2 | *wbpL* | 2223..3266 | 47.03 | Glycos_transf_4; PF00953.17; E value=7.2e-29 | polyprenol phosphate:N-acetyl-hexosamine 1-phosphate transferase [Yersinia frederiksenii] (CNF58510.1) | 53/70(347) | polyprenol phosphate:N-acetyl-hexosamine 1-phosphate transferase |
| 3 | *AT* | 3259..3819 | 46.88 | Hexapep; PF00132.20; E value=4.9e-08 | sugar O-acyltransferase [Enterobacter cloacae complex sp. 35669] (WP_045260407.1) | 99/99(186) | UDP-N-acetylbacillosamine N-acetyltransferase |
| 4 | *wbpM* | 3858..5744 | 46.58 | Polysacc_synt_2; PF02719.11; E value=2e-127 | nucleoside-diphosphate sugar epimerase [Enterobacter cloacae complex sp. 35669] (KJN34375.1) | 98/98(628) | nucleoside-diphosphate sugarepimerase/dehydratase |
| 5 | *rmlB* | 5847..6932 | 47.42 | GDP_Man_Dehyd; PF16363.1; E value=7e-99 | dTDP-glucose 4,6-dehydratase [Enterobacter cloacae] (WP_063162895.1) | 95/97(361) | dTDP-glucose 4,6-dehydratase |
| 6 | *rmlD* | 6932..7831 | 50.78 | RmlD_sub_bind; PF04321.13; E value=8.7e-107 | dTDP-4-dehydrorhamnose reductase [Escherichia coli] (WP_001023628.1) | 87/93(299) | dTDP-4-dehydrorhamnose reductase |
| 7 | *rmlA* | 7884..8762 | 44.14 | NTP_transferase; PF00483.19; E value=3.1e-74 | glucose-1-phosphate thymidylyltransferase RfbA [Enterobacter cloacae] (WP_063923190.1) | 98/98(292) | Glucose-1-phosphate thymidylyltransferas |
| 8 | *rmlC* | 8766..9314 | 37.7 | dTDP_sugar_isom; PF00908.13; E value=4.8e-77 | RmlC [Escherichia coli] (ACD37177.1) | 77/87(182) | dTDP-4-dehydrorhamnose 3,5-epimerase |
| 9 | *wzx* | 9455..10597 | 33.95 | Polysacc_synt; PF01943.13; E value=6.5e-41 | flippase [Raoultella ornithinolytica] (WP_086816156.1) | 38/61(380) | O-antigen flippase |
| 10 | *wzy* | 10594..11760 | 31.71 | PerB; PF06590.7; E value=0.049 | Wzy [Shigella boydii CDC 3083-94] (ACD06325.1) | 44/62(388) | polymerase |
| 11 | *GT1* | 11757..12851 | 33.42 | Glyco_tran_WbsX; PF14307.2; E value=1.1e-120 | glycosyl transferase [Acinetobacter sp. ANC 4973] (WP_086185218.1) | 55/70(364) | glycosyl transferas |
| 12 | *GT2* | 12858..13727 | 37.36 | Glycos_transf_2; PF00535.22; E value=8.2e-12 | rhamnosyltransferase [Enterobacter cloacae] (WP_063154997.1) | 96/97(289) | Glycosyl transferase family 2 |
| 13 | *GT3* | 13802..14914 | 38.27 | Glycos_transf_1; PF00534.16; E value=4.7e-42 | group 1 glycosyl transferase [Enterobacter cloacae] (SAD52852.1) | 88/94(370) | glycosyltransferase |

| *E. cloacae* O13 | | | | | | | |
| --- | --- | --- | --- | --- | --- | --- | --- |
| Orf no. | Gene name | Position of gene | G+C content(%) | Conserved domain(s) | Similar protein(s), strain(s) (Genbank accession No.) | %Identical/%Similar (total No. of aa) | Putative function of protein |
| 1 | *rmlB* | 1256..2341 | 51.57 | GDP_Man_Dehyd; PF16363.1; E value=4.3e-98 | dTDP-glucose 4,6-dehydratase [Enterobacter hormaechei] (WP_045338357.1) | 99/99(361) | dTDP-glucose 4,6-dehydratase |
| 2 | *rmlD* | 2341..3240 | 51.56 | RmlD_sub_bind; PF04321.13; E value=1.1e-108 | dTDP-4-dehydrorhamnose reductase [Enterobacter cloacae] (WP_047637007.1) | 98/99(299) | dTDP-4-dehydrorhamnose reductase |
| 3 | *rmlA* | 3293..4171 | 50.28 | NTP_transferase; PF00483.19; E value=1e-73 | glucose-1-phosphate thymidylyltransferase RfbA [Enterobacter cloacae] (WP_063151865.1) | 99/100(292) | Glucose-1-phosphate thymidylyltransferase |
| 4 | *rmlC* | 4175..4711 | 40.04 | dTDP_sugar_isom; PF00908.13; E value=4.7e-79 | dTDP-4-dehydrorhamnose 3,5-epimerase [Enterobacter cloacae] (WP_063158410.1) | 90/93(178) | dTDP-4-dehydrorhamnose 3,5-epimerase |
| 5 | *GT1* | 4822..5607 | 30.79 | DUF616; PF04765.9; E value=2.1e-17 | glycosyl transferase [Sulfurimonas sp.] (PLY14356.1) | 42/62(261) | glycosyl transferase |
| 6 | *wzy* | 5617..6627 | 29.08 | EpsG; PF14897.2; E value=4.4e-41 | O-antigen and lipid-linked capsular repeat unit polymerase [Klebsiella sp. 1754/49] (BAT23399.1) | 34/53(336) | O-antigen polymerase |
| 7 | *GT2* | 6639..7526 | 31.64 | Glycos_transf_2; PF00535.22; E value=1.1e-15 | rhamnosyltransferase [Enterobacter cloacae] (WP_032671726.1) | 96/98(295) | Glycosyl transferase family 2 |
| 8 | *GT3* | 7573..8352 | 29.36 | Glycos_transf_2; PF00535.22; E value=0.015 | glycosyl transferase family 2 [Enterobacter hormaechei subsp. steigerwaltii] (KJP72838.1) | 94/97(259) | Rhamnosyltransferase WbbL |

| *E. cloacae* O14 | | | | | | | |
| --- | --- | --- | --- | --- | --- | --- | --- |
| Orf no. | Gene name | Position of gene | G+C content(%) | Conserved domain(s) | Similar protein(s), strain(s) (Genbank accession No.) | %Identical/%Similar (total No. of aa) | Putative function of protein |
| 1 | *pseB* | 1284..2282 | 44.44 | Polysacc_synt_2; PF02719.11; E value=1.7e-100 | UDP-N-acetylglucosamine 4,6-dehydratase [Shigella boydii ATCC 9905] (EFW52883.1) | 95/96(332) | UDP-N-acetylglucosamine 4,6-dehydratase |
| 2 | *pesC* | 2282..3433 | 48.35 | DegT_DnrJ_EryC1; PF01041.13; E value=1.7e-110 | UDP-4-amino-4,6-dideoxy-N-acetyl-beta-L-altrosamine transaminase [Escherichia coli] (WP_063501742.1) | 83/90(383) | UDP-4-amino-4, 6-dideoxy-N-acetyl-beta-L-altrosamine transaminase |
| 3 | *pseF* | 3434..4144 | 44.44 | CTP_transf_3; PF02348.15; E value=1.5e-33 | pseudaminic acid cytidylyltransferase [Escherichia coli] (WP_054472859.1) | 79/90(236) | CMP-N,N'-diacetyllegionaminic acid synthase |
| 4 | *pseG/H* | 4122..5657 | 40.69 | Glyco_trans_1_3; PF13528.2; E value=8.9e-21 | UDP-2,4-diacetamido-2,4,6-trideoxy-beta-L-altropyranose hydrolase [Escherichia coli] (WP_063122236.1) | 85/92(511) | UDP-2,4-diacetamido-2,4, 6-trideoxy-beta-L-altropyranose hydrolase |
| 5 | *pesI* | 5654..6703 | 42.1 | NeuB; PF03102.10; E value=4.1e-90 | pseudaminic acid synthase [Escherichia coli] (WP_096840056.1) | 83/92(349) | Pseudaminic acid synthase |
| 6 | *wzx* | 6745..8010 | 30.25 |  | O-antigen flippase [Escherichia coli] (AIG62417.1) | 33/54(421) | flippase |
| 7 | *orf7* | 7985..9382 | 33.33 | Capsule_synth; PF05159.10; E value=5e-10 | hypothetical protein [Enterobacter cloacae] (WP_088244291.1) | 89/94(465) | hypothetical protein |
| 8 | *wzy* | 9383..10708 | 34.69 |  | Wzy [Shigella boydii] (ACD37072.1) | 25/44(441) | polymerase |
| 9 | *GT1* | 10701..11669 | 31.58 | Glycos_transf_2; PF00535.22; E value=7.8e-26 | glycosyltransferase family 2 protein [Enterobacter cloacae] (WP_058683796.1) | 35/54(322) | putative glycosyltransferase |
| 10 | *GT2* | 11729..12493 | 40.52 | DUF4422; PF14393.2; E value=4.9e-76 | glycosyltransferase [Klebsiella pneumoniae] (SLW02940.1) | 44/63(254) | putative glycosyltransferase |
| 11 | *glf* | 12490..13635 | 43.11 | GLF; PF03275.9; E value=2.5e-76 | UDP-galactopyranose mutase [Citrobacter koseri] (WP_012131729.1) | 92/96(381) | UDP-galactopyranose mutase |
| 12 | *wbaP* | 13650..15086 | 41.96 | Bac_transf; PF02397.12; E value=3.2e-57 | galactosyl-1-phosphate transferase [Salmonella enterica] (AAC44096.1) | 78/89(478) | undecaprenyl-phosphate galactosephosphotransferase |

| *E. cloacae* O15 | | | | | | | |
| --- | --- | --- | --- | --- | --- | --- | --- |
| Orf no. | Gene name | Position of gene | G+C content(%) | Conserved domain(s) | Similar protein(s), strain(s) (Genbank accession No.) | %Identical/%Similar (total No. of aa) | Putative function of protein |
| 1 | *rmlB* | 1257..2342 | 50.92 | GDP_Man_Dehyd; PF16363.1; E value=4.3e-99 | dTDP-glucose 4,6-dehydratase [Enterobacter cloacae] (WP_063151864.1) | 98/99(361) | dTDP-glucose 4,6-dehydratase |
| 2 | *rmlD* | 2342..3241 | 51.44 | RmlD_sub_bind; PF04321.13; E value=2.8e-108 | dTDP-4-dehydrorhamnose reductase [Enterobacter cloacae] (WP_063855582.1) | 99/99(299) | dTDP-4-dehydrorhamnose reductase |
| 3 | *rmlA* | 3294..4172 | 50.74 | NTP_transferase; PF00483.19; E value=1.4e-73 | glucose-1-phosphate thymidylyltransferase RfbA [Enterobacter cloacae] (WP_062728858.1) | 98/99(292) | Glucose-1-phosphate thymidylyltransferase |
| 4 | *rmlC* | 4176..4724 | 38.07 | dTDP_sugar_isom; PF00908.13; E value=6.5e-77 | dTDP-4-dehydrorhamnose 3,5-epimerase [Escherichia coli] (WP_097317000.1) | 88/93(182) | dTDP-4-dehydrorhamnose 3,5-epimerase |
| 5 | *orf5* | 4729..5712 | 32.42 | Nitroreductase; PF00881.20; E value=1.6e-17 | hypothetical protein [Escherichia coli] (WP_097316999.1) | 73/87(327) | hypothetical protein |
| 6 | *wzx* | 5745..7247 | 29.87 | Polysacc_synt; PF01943.13; E value=1.5e-06 | Wzx [Shigella dysenteriae] (ACA24787.1) | 75/87(500) | putative membrane protein EpsK |
| 7 | *PT* | 7250..8404 | 32.64 | PS_pyruv_trans; PF04230.9; E value=2.1e-33 | polysaccharide pyruvyl transferase family protein [Escherichia coli] (WP_096998569.1) | 45/64(384) | Polysaccharide pyruvyl transferase |
| 8 | *GT1* | 8415..9308 | 30.65 | Glycos_transf_2; PF00535.22; E value=9.2e-10 | glycosyltransferase family 2 protein [Photobacterium sp. CECT 9192] (WP_087853898.1) | 54/70(297) | Glycosyl transferase family 2 |
| 9 | *GT2* | 9295..10200 | 33.22 | Glycos_transf_2; PF00535.22; E value=2.3e-12 | rhamnosyltransferase [Escherichia coli] (WP_098027495.1) | 48/63(301) | Glycosyl transferase family 2 |
| 10 | *GT3* | 10211..11095 | 34.12 | Glycos_transf_2; PF00535.22; E value=8.6e-12 | glycosyl transferase [Escherichia coli] (glycosyl transferase [Escherichia coli]) | 72/87(294) | Putative glycosyltransferase EpsE |
| 11 | *wzy* | 11114..12211 | 31.33 | EpsG; PF14897.2; E value=4.8e-20 | O69 family O-antigen polymerase [Escherichia coli] (WP_032187476.1) | 31/51(365) | O-antigen polymerase |

| *E. cloacae* O16 | | | | | | | |
| --- | --- | --- | --- | --- | --- | --- | --- |
| Orf no. | Gene name | Position of gene | G+C content(%) | Conserved domain(s) | Similar protein(s), strain(s) (Genbank accession No.) | %Identical/%Similar (total No. of aa) | Putative function of protein |
| 1 | *rmlB* | 1201..2334 | 48.85 | GDP_Man_Dehyd; PF16363.1; E value=3e-98 | dTDP-glucose 4,6-dehydratase [Enterobacter cloacae]( WP_061097683.1) | 99/99(377) | dTDP-glucose 4,6-dehydratase |
| 2 | *wbpK* | 2389..3333 | 41.06 | Epimerase; PF01370.17; E value=1.8e-29 | NAD-dependent epimerase [Enterobacter cloacae complex sp. GN02283] (WP_047363173.1) | 97/99(314) | NAD-dependent epimerase/dehydratase |
| 3 | *wbpL* | 3333..4367 | 41.93 | Glycos_transf_4; PF00953.17; E value=4.7e-29 | polyprenol phosphate:N-acetyl-hexosamine 1-phosphate transferase [Yersinia frederiksenii] (CFQ40598.1) | 52/71(344) | polyprenol phosphate:N-acetyl-hexosamine 1-phosphate transferase |
| 4 | *AT* | 4368..4928 | 44.39 | Hexapep; PF00132.20; E value=1.2e-06 | sugar O-acyltransferase [Enterobacter hormaechei] (WP_096217051.1) | 94/96(186) | UDP-N-acetylbacillosamine N-acetyltransferase |
| 5 | *wbpM* | 4967..6868 | 43.22 | Polysacc_synt_2; PF02719.11; E value=1.6e-126 | nucleoside-diphosphate sugar epimerase/dehydratase [Yokenella regensburgei ATCC 49455] (KFD21689.1) | 91/95(633) | nucleoside-diphosphate sugar epimerase/dehydratase |
| 6 | *rmlC* | 6923..7462 | 39.44 | dTDP_sugar_isom; PF00908.13; E value=7.8e-72 | dTDP-4-dehydrorhamnose 3,5-epimerase [Enterobacter cloacae] (WP_094934028.1) | 67/82(179) | dTDP-4-dehydrorhamnose 3,5-epimerase |
| 7 | *tll* | 7459..8277 | 37.61 | Epimerase; PF01370.17; E value=1.2e-20 | Tll [Aeromonas piscicola] (ABX39499.1) | 56/69(272) | dTDP-6-deoxy-L-xylo-hexulose reductase |
| 8 | *wzx* | 8270..9529 | 36.51 | DUF3976; PF13121.2; E value=4 | O45 family O-antigen flippase, partial [Escherichia coli] (WP_089537930.1) | 91/95(419) | flippase |
| 9 | *GT1* | 9516..10490 | 39.08 | Glycos_transf_2; PF00535.22; E value=5.4e-21 | putative glycosyl transferase [Escherichia coli S88] (CAN87667.1) | 39/55(324) | glycosyl transferase |
| 10 | *wzy* | 10492..11640 | 36.03 | Crinivirus_P26; PF07416.7; E value=0.01 | O-antigen polysaccharide polymerase Wzy [Spirosoma fluviale] (WP_097129000.1) | 25/46(382) | O-antigen polysaccharide polymerase |
| 11 | *cysE* | 11633..12169 | 36.87 | Hexapep; PF00132.20; E value=7.7e-09 | serine acetyltransferase [Collimonas fungivorans] (WP_041741248.1) | 44/70(178) | Serine acetyltransferase |
| 12 | *orf12* | 12171..13181 | 33.33 | DUF3880; PF12996.3; E value=0.0051 | hypothetical protein [Escherichia coli] (WP_000865877.1) | 88/94(336) | hypothetical protein |
| 13 | *rmlA* | 13230..14102 | 41.7 | NTP_transferase; PF00483.19; E value=1.7e-68 | glucose-1-phosphate thymidylyltransferase [Yokenella regensburgei] (WP_038256531.1) | 90/95(290) | Glucose-1-phosphate thymidylyltransferase |
| 14 | *orf14* | 14161..15627 | 37.36 | Glyco_hydro_1; PF00232.14; E value=4.7e-120 | hypothetical protein [Enterobacter sp. DC1] (WP_084492147.1) | 91/95(488) | hypothetical protein |

| *E. cloacae* O17 | | | | | | | |
| --- | --- | --- | --- | --- | --- | --- | --- |
| Orf no. | Gene name | Position of gene | G+C content(%) | Conserved domain(s) | Similar protein(s), strain(s) (Genbank accession No.) | %Identical/%Similar (total No. of aa) | Putative function of protein |
| 1 | *wzx* | 1368..2465 | 34.34 | Polysacc_synt; PF01943.13; E value=9e-48 | flippase [Budvicia aquatica] (WP_084167195.1) | 63/81(365) | Putative O-antigen transporter |
| 2 | *glf* | 2478..3575 | 35.88 | GLF; PF03275.9; E value=4.8e-85 | UDP-galactopyranose mutase [Hafnia alvei] (ANF30175.1) | 76/85(365) | UDP-galactopyranose mutase |
| 3 | *GT1* | 3596..4588 | 32.33 | Glyco_trans_1_4; PF13692.2; E value=0.023 | beta-1,6-galactofuranosyltransferase [Escherichia coli XH140A] (EGU24932.1) | 38/58(330) | Beta-1,6-galactofuranosyltransferase WbbI |
| 4 | *wzy* | 4621..5796 | 31.04 | O-antigen_lig; PF13425.2; E value=0.00015 | O-antigen polymerase [Streptococcus sp. DD11] (KXT85152.1) | 26/47(391) | O-antigen polymerase |
| 5 | *GT2* | 5796..6902 | 35.32 | Glycos_transf_1; PF00534.16; E value=5.1e-27 | glycosyltransferase family 1 protein [Vibrio anguillarum] (WP_013855789.1) | 53/71(368) | glycosyltransferase family 1 protein |

| *E. cloacae* O18 | | | | | | | |
| --- | --- | --- | --- | --- | --- | --- | --- |
| Orf no. | Gene name | Position of gene | G+C content(%) | Conserved domain(s) | Similar protein(s), strain(s) (Genbank accession No.) | %Identical/%Similar (total No. of aa) | Putative function of protein |
| 1 | *rmlB* | 1250..2335 | 51.38 | GDP_Man_Dehyd; PF16363.1; E value=8.6e-98 | dTDP-glucose 4,6-dehydratase [Enterobacter kobei] (WP_047027448.1) | 98/98(361) | dTDP-glucose 4,6-dehydratase |
| 2 | *rmlD* | 2335..3234 | 51.89 | RmlD_sub_bind; PF04321.13; E value=7.3e-109 | dTDP-4-dehydrorhamnose reductase [Enterobacter ludwigii] (WP_047355742.1) | 98/98(299) | dTDP-4-dehydrorhamnose reductase |
| 3 | *rmlA* | 3287..4165 | 48.24 | NTP_transferase; PF00483.19; E value=1.3e-73 | glucose-1-phosphate thymidylyltransferase RfbA [Escherichia coli] (WP_001376204.1) | 98/99(292) | Glucose-1-phosphate thymidylyltransferase |
| 4 | *rmlC* | 4169..4717 | 37.89 | dTDP_sugar_isom; PF00908.13; E value=8.9e-75 | RmlC [Cronobacter turicensis] (AGJ50607.1) | 73/84(182) | dTDP-4-dehydrorhamnose 3,5-epimerase |
| 5 | *fdtA* | 4720..5139 | 39.05 | FdtA; PF05523.7; E value=1.3e-51 | dTDP-6-deoxy-3,4-keto-hexulose isomerase [Cronobacter turicensis] (WP_105582371.1) | 81/92(139) | TDP-4-oxo-6-deoxy-alpha-D-glucose-3, 4-oxoisomerase |
| 6 | *fdtC* | 5111..5584 | 37.13 | Hexapep; PF00132.20; E value=2.3e-23 | FdtC [Cronobacter muytjensii] (AEH27511.1) | 78/88(157) | dTDP-3-amino-3,6-dideoxy-alpha-D-galactopyranose 3-N-acetyltransferase |
| 7 | *fdtB* | 5574..6680 | 38.39 | DegT_DnrJ_EryC1; PF01041.13; E value=5e-112 | DegT/DnrJ/EryC1/StrS family aminotransferase [Kosakonia cowanii] (WP_076770124.1) | 73/84(368) | dTDP-3-amino-3,6-dideoxy-alpha-D-galactopyranose transaminase |
| 8 | *wzx* | 6677..7960 | 36.21 | Polysacc_synt; PF01943.13; E value=3.9e-07 | O-antigen flippase [Cronobacter dublinensis] (WP_105639466.1) | 49/72(427) | flippase |
| 9 | *GT1* | 7944..9356 | 35.95 |  | glycosyl transferase, partial [Cronobacter sakazakii] (WP_105573592.1) | 48/68(470) | glycosyl transferase |
| 10 | *wzy* | 9360..10673 | 32.19 | PIG-F; PF06699.7; E value=0.012 | O70 family O-antigen polymerase [Escherichia coli] (WP_001752773.1) | 24/43(437) | O-antigen polymerase |
| 11 | *GT2* | 10683..11732 | 34.76 | Glycos_transf_2; PF00535.22; E value=3.1e-08 | glycosyltransferase family 2 protein [Kosakonia cowanii] (WP_076770120.1) | 57/74(349) | glycosyl transferase family 2 |
| 12 | *GT3* | 11736..12821 | 36.65 | Glycos_transf_1; PF00534.16; E value=1.2e-26 | glycosyltransferase [Kosakonia cowanii] (WP_076770119.1) | 53/73(361) | glycosyltransferase |
| 13 | *GT4* | 12823..13593 | 34.5 | Glycos_transf_2; PF00535.22; E value=1.9e-31 | glycosyltransferase family 2 protein [Kosakonia cowanii] (WP_076770118.1) | 72/81(256) | glycosyltransferase TuaG |

| *E. cloacae* O19 | | | | | | | |
| --- | --- | --- | --- | --- | --- | --- | --- |
| Orf no. | Gene name | Position of gene | G+C content(%) | Conserved domain(s) | Similar protein(s), strain(s) (Genbank accession No.) | %Identical/%Similar (total No. of aa) | Putative function of protein |
| 1 | *wzy* | 1232..2506 | 34.67 | WzyE; PF06899.7; E value=4.7e-12 | O-antigen assembly polymerase [Chelonobacter oris] (WP_034617974.1) | 25/45(424) | O-antigen polymerase |
| 2 | *GT1* | 2484..3545 | 39.08 | Glycos_transf_1; PF00534.16; E value=5.1e-29 | glycosyltransferase family 1 protein [Vibrio campbellii] (WP_081234210.1) | 38/58(353) | glycosyltransferase family 1 protein |
| 3 | *orf3* | 3536..4756 | 35.3 |  | hypothetical protein [Enterobacter hormaechei] (WP_047056184.1) | 27/48(406) | hypothetical protein |
| 4 | *GT2* | 4740..5240 | 36.33 | Alg14; PF08660.7; E value=4.6e-17 | UDP-N-acetylglucosamine transferase [Clostridium chromiireducens] (OPJ59166.1) | 30/50(166) | UDP-N-acetylglucosamine transferase |
| 5 | *GT3* | 5237..5704 | 35.04 | Glyco_tran_28_C; PF04101.12; E value=9.2e-18 | glycosyl transferase [Vibrio parahaemolyticus] (WP_025500312.1) | 45/63(155) | glycosyl transferase |

| *E. cloacae* O20 | | | | | | | |
| --- | --- | --- | --- | --- | --- | --- | --- |
| Orf no. | Gene name | Position of gene | G+C content(%) | Conserved domain(s) | Similar protein(s), strain(s) (Genbank accession No.) | %Identical/%Similar (total No. of aa) | Putative function of protein |
| 1 | *rmlB* | 1255..2340 | 51.1 | GDP_Man_Dehyd; PF16363.1; E value=5.2e-98 | dTDP-glucose 4,6-dehydratase [Enterobacter cloacae] (WP_063962828.1) | 99/99(361) | dTDP-glucose 4,6-dehydratase |
| 2 | *rmlD* | 2340..3239 | 50.89 | RmlD_sub_bind; PF04321.13; E value=3.3e-108 | dTDP-4-dehydrorhamnose reductase [Enterobacter hormaechei] (WP_059371000.1) | 98/99(299) | dTDP-4-dehydrorhamnose reductase |
| 3 | *rmlA* | 3292..4170 | 49.6 | NTP_transferase; PF00483.19; E value=2.6e-73 | glucose-1-phosphate thymidylyltransferase RfbA [Escherichia coli] (WP_001376204.1) | 98/99(292) | Glucose-1-phosphate thymidylyltransferase |
| 4 | *rmlC* | 4174..4710 | 39.85 | dTDP_sugar_isom; PF00908.13; E value=4.4e-78 | dTDP-4-dehydrorhamnose 3,5-epimerase [Enterobacter cloacae] (WP_014170804.1) | 87/91(178) | dTDP-4-dehydrorhamnose 3,5-epimerase |
| 5 | *wzx* | 4758..5885 | 30.76 |  | Wzx [Shigella boydii] (AAV41070.1) | 58/75(375) | flippase |
| 6 | *PT* | 5878..6714 | 35.72 | PS_pyruv_trans; PF04230.9; E value=1.1e-13 | polysaccharide pyruvyl transferase family protein [Thalassotalea agarivorans] (WP_093326989.1) | 48/67(278) | Exopolysaccharide glucosyl ketal-pyruvate-transferas |
| 7 | *wzy* | 6743..7798 | 29.36 | EpsG; PF14897.2; E value=4.1e-19 | O-antigen polymerase [Shigella boydii] (WP_073692825.1) | 57/73(351) | O-antigen polymerase |
| 8 | *GT1* | 7795..8613 | 32.36 | Glycos_transf_2; PF00535.22; E value=6.1e-24 | glycosyltransferase family 2 protein (WP_053290776.1) | 61/76(272) | glycosyltransferase family 2 protein |
| 9 | *GT2* | 8647..9729 | 35.09 | DUF1972; PF09314.7; E value=2.1e-29 | glycosyltransferase family 1 protein [Enterobacter ludwigii] (WP_047355738.1) | 84/92(360) | glycosyl transferase |

| *E. cloacae* O21 | | | | | | | |
| --- | --- | --- | --- | --- | --- | --- | --- |
| Orf no. | Gene name | Position of gene | G+C content(%) | Conserved domain(s) | Similar protein(s), strain(s) (Genbank accession No.) | %Identical/%Similar (total No. of aa) | Putative function of protein |
| 1 | *pseB* | 1273..2271 | 43.84 | Polysacc_synt_2; PF02719.11; E value=2.7e-100 | UDP-N-acetylglucosamine 4,6-dehydratase [Shigella boydii ATCC 9905] (EFW52883.1) | 95/96(332) | UDP-N-acetylglucosamine 4,6-dehydratase |
| 2 | *pesC* | 2271..3422 | 46.96 | DegT_DnrJ_EryC1; PF01041.13; E value=7.4e-112 | UDP-4-amino-4,6-dideoxy-N-acetyl-beta-L-altrosamine transaminase [Escherichia coli] (WP_063501742.1) | 83/90(383) | UDP-4-amino-4, 6-dideoxy-N-acetyl-beta-L-altrosamine transaminase |
| 3 | *pseF* | 3423..4133 | 45.43 | CTP_transf_3; PF02348.15; E value=1.6e-33 | pseudaminic acid cytidylyltransferase [Escherichia coli] (WP_054472859.1) | 79/90(236) | CMP-N,N'-diacetyllegionaminic acid synthase |
| 4 | *pseG/H* | 4111..5643 | 40.77 | Glyco_trans_1_3; PF13528.2; E value=3.2e-21 | UDP-2,4-diacetamido-2,4,6-trideoxy-beta-L-altropyranose hydrolase [Escherichia coli] (WP_063122236.1) | 85/92(510) | UDP-2,4-diacetamido-2,4, 6-trideoxy-beta-L-altropyranose hydrolase |
| 5 | *pesI* | 5643..6692 | 42.1 | NeuB; PF03102.10; E value=1.8e-90 | pseudaminic acid synthase [Escherichia coli] (WP_096840056.1) | 83/92(349) | Pseudaminic acid synthase |
| 6 | *wzx* | 6699..7904 | 32.34 | Polysacc_synt; PF01943.13; E value=1.5e-20 | flippase [Acinetobacter indicus] (WP_075167655.1) | 42/65(401) | flippase |
| 7 | *GT1* | 7901..8848 | 30.27 | Glyco_transf_52; PF07922.7; E value=6.6e-33 | beta-galactosamide-alpha-2,3-sialyltransferase [Kosakonia oryzae] (WP_079517680.1) | 58/77(315) | beta-galactosamide-alpha-2,3-sialyltransferase |
| 8 | *wzy* | 8876..10198 | 33.18 |  | Wzy [Shigella boydii] (ACD37072.1) | 32/48(440) | polymerase |
| 9 | *GT2* | 10188..11162 | 32.51 | Glycos_transf_2; PF00535.22; E value=3.9e-29 | glycosyltransferase family 2 protein [Enterobacter cloacae] (WP_058683796.1) | 56/73(324) | putative glycosyltransferase |
| 10 | *orf10* | 11166..11936 | 40.86 | DUF4422; PF14393.2; E value=1.1e-77 | DUF4422 domain-containing protein [Escherichia coli] (WP_063122230.1) | 91/93(256) | hypothetical protein |
| 11 | *glf* | 11933..13078 | 44.85 | GLF; PF03275.9; E value=1.1e-76 | UDP-galactopyranose mutase [Citrobacter koseri] (WP_012131729.1) | 94/97(381) | UDP-galactopyranose mutase |
| 12 | *wbaP* | 13093..14529 | 42.52 | Bac_transf; PF02397.12; E value=6.6e-57 | undecaprenyl-phosphate galactose phosphotransferase WbaP [Salmonella enterica] (WP_061423802.1) | 78/90(478) | undecaprenyl-phosphate galactosephosphotransferase |

| *E. cloacae* O22 | | | | | | | |
| --- | --- | --- | --- | --- | --- | --- | --- |
| Orf no. | Gene name | Position of gene | G+C content(%) | Conserved domain(s) | Similar protein(s), strain(s) (Genbank accession No.) | %Identical/%Similar (total No. of aa) | Putative function of protein |
| 1 | *GT1* | 1223..2035 | 30.87 | Glyco_transf_25; PF01755.13; E value=0.04 | glycosyltransferase family 2 protein [Vibrio maritimus]( WP_081936136.1) | 33/52(269) | glycosyltransferase family 2 |
| 2 | *wzy* | 2032..3261 | 30.33 | EpsG; PF14897.2; E value=2.8e-16 | Wzy [Shigella dysenteriae] (ACA24776.1) | 54/74(409) | O-antigen polymerase |
| 3 | *wzx* | 3264..4571 | 32.34 | Polysacc_synt_C; PF14667.2; E value=9.1e-10 | flippase [Erwinia iniecta] (WP_052897555.1) | 49/71(437) | flippase |
| 4 | *GT2* | 4556..5422 | 30.8 | Glycos_transf_2; PF00535.22; E value=5.8e-24 | glycosyltransferase family 2 protein [Enterobacter cloacae] (WP_062938702.1) | 69/82(288) | putative glycosyltransferase EpsJ |
| 5 | *GT3* | 5419..6105 | 33.92 | Gly_transf_sug; PF04488.11; E value=2.2e-12 | tcdA/TcdB catalytic glycosyltransferase, partial [Klebsiella pneumoniae] (WP_094018886.1) | 37/56(228) | glycosyltransferase |
| 6 | *galE* | 6107..7120 | 37.08 | GDP_Man_Dehyd; PF16363.1; E value=8e-57 | UDP-glucose 4-epimerase GalE [Escherichia coli] (WP_104773228.1) | 66/80(337) | UDP-glucose 4-epimerase |
| 7 | *GT4* | 7134..7880 | 31.73 | Glycos_transf_2; PF00535.22; E value=2.6e-31 | glycosyltransferase family 2 protein [Escherichia coli] (WP_047623705.1) | 65/82(248) | glycosyltransferase |

| *E. cloacae* O23 | | | | | | | |
| --- | --- | --- | --- | --- | --- | --- | --- |
| Orf no. | Gene name | Position of gene | G+C content(%) | Conserved domain(s) | Similar protein(s), strain(s) (Genbank accession No.) | %Identical/%Similar (total No. of aa) | Putative function of protein |
| 1 | *rmlB* | 1297..2385 | 49.13 | GDP_Man_Dehyd; PF16363.1; E value=5.7e-99 | dTDP-glucose 4,6-dehydratase [Kosakonia sacchari] (WP_065370578.1) | 88/95(362) | dTDP-glucose 4,6-dehydratase |
| 2 | *rmlA* | 2400..3266 | 40.6 | NTP_transferase; PF00483.19; E value=2.1e-67 | glucose-1-phosphate thymidylyltransferase [Escherichia coli] (WP_032180400.1) | 86/93(288) | glucose-1-phosphate thymidylyltransferase |
| 3 | *orf3* | 3310..3639 | 42.12 | Cupin_2; PF07883.7; E value=0.025 | MULTISPECIES: hypothetical protein [Pantoea] (WP_033758738.1) | 78/89(109) | hypothetical protein |
| 4 | *GT1* | 3639..4394 | 36.38 | NTP_transferase; PF00483.19; E value=2.2e-11 | glycosyl transferase family 2 [Pantoea agglomerans] (WP_033767808.1) | 70/81(251) | glycosyl transferase family 2 |
| 5 | *GT2* | 4397..6043 | 34.18 | Glycos_transf_1; PF00534.16; E value=3.1e-18 | glycosyl transferase [Myxococcus fulvus] (WP_046714718.1) | 25/39(548) | UDP-D-galactose:(glucosyl)lipopolysaccharide-1, 6-D-galactosyltransferase |
| 6 | *wavE* | 6040..7056 | 32.15 | WavE; PF07507.7; E value=1.4e-10 | WavE lipopolysaccharide synthesis (SFA69232.1) | 32/53(338) | lipopolysaccharide synthesis |
| 7 | *fcf1* | 7043..7981 | 37.06 | GDP_Man_Dehyd; PF16363.1; E value=1.4e-31 | dTDP-4-dehydro-6-deoxyglucose reductase (Q6E7F2.1) | 31/44(312) | reductase |
| 8 | *wavE* | 8011..9069 | 32.39 | WavE; PF07507.7; E value=3.5e-41 | WavE lipopolysaccharide synthesis [Hafnia sp. HMSC23F03] (WP_070715892.1) | 41/57(352) | lipopolysaccharide synthesis |
| 9 | *wzm* | 9073..9891 | 35.16 | ABC2_membrane; PF01061.20; E value=1.6e-18 | ABC transporter permease [Pantoea rwandensis] (WP_038645992.1) | 72/86(272) | permease |
| 10 | *wzt* | 9888..10619 | 36.61 | ABC_tran; PF00005.23; E value=4.3e-19 | ABC transporter ATP-binding protein [Pantoea agglomerans] (WP_033788287.1) | 69/87(243) | ABC transporter ATP-binding protein |
| 11 | *orf11* | 10655..11257 | 32.67 |  | hypothetical protein [Xenorhabdus sp. KJ12.1] (WP_099111864.1) | 60/78(200) | hypothetical protein |
| 12 | *orf12* | 11250..11894 | 35.66 | HAD_2; PF13419.2; E value=5.8e-20 | HAD family phosphatase [Xenorhabdus sp. KJ12.1] (WP_099111865.1) | 80/90(241) | phosphatase |
| 13 | *GT3* | 11891..12958 | 36.8 | SnoaL_2; PF12680.3; E value=4e-11 | glycosyl transferase family 2 [Campylobacter sp. RM8964] (WP_086333328.1) | 37/62(355) | glycosyl transferase |

| *E. cloacae* O24 | | | | | | | |
| --- | --- | --- | --- | --- | --- | --- | --- |
| Orf no. | Gene name | Position of gene | G+C content(%) | Conserved domain(s) | Similar protein(s), strain(s) (Genbank accession No.) | %Identical/%Similar (total No. of aa) | Putative function of protein |
| 1 | *orf1* | 1042..1677 | 34.28 | HAD; PF12710.3; E value=3.2e-05 | hypothetical protein [Escherichia coli] (WP_071342959.1) | 40/61(211) | hypothetical protein |
| 2 | *orf2* | 1625..2479 | 26.9 |  | hypothetical protein [Escherichia coli] (WP_089530828.1) | 49/67(284) | hypothetical protein |
| 3 | *wzy* | 2483..3787 | 29.12 | O-ag_pol_Wzy; PF14296.2; E value=4.3e-09 | O-antigen polysaccharide polymerase Wzy [Escherichia coli] (WP_057696920.1) | 60/79(434) | O-antigen polysaccharide polymerase |
| 4 | *wzx* | 3789..4997 | 30.85 | Polysacc_synt_3; PF13440.2; E value=6.9e-06 | O antigen flippase [Pseudomonas aeruginosa] (WP_003150881.1) | 24/49(402) | O antigen flippase |
| 5 | *GT1* | 4990..6081 | 29.95 | Glycos_transf_1; PF00534.16; E value=5.6e-31 | glycosyltransferase [Escherichia coli] (WP_057687839.1) | 53/70(363) | glycosyltransferase |
| 6 | *GT2* | 6056..6895 | 33.93 | Glycos_transf_2; PF00535.22; E value=1.2e-17 | glycosyl transferase [Providencia alcalifaciens] (WP_006663209.1) | 51/71(279) | glycosyltransferase |

| *E. cloacae* O26 | | | | | | | |
| --- | --- | --- | --- | --- | --- | --- | --- |
| Orf no. | Gene name | Position of gene | G+C content(%) | Conserved domain(s) | Similar protein(s), strain(s) (Genbank accession No.) | %Identical/%Similar (total No. of aa) | Putative function of protein |
| 1 | *wzx* | 1312..2706 | 32.47 | Polysacc_synt; PF01943.13; E value=3.4e-12 | O84 family O-antigen flippase [Escherichia coli] (WP_062893556.1) | 63/80(464) | flippase |
| 2 | *AT* | 2707..3318 | 34.8 | Hexapep; PF00132.20; E value=6.5e-13 | acyltransferase [Escherichia albertii] (WP_105228546.1) | 75/88(203) | acyltransferase |
| 3 | *galE* | 3338..4243 | 34.0 | Epimerase; PF01370.17; E value=1.8e-35 | UDP-glucose 4-epimerase [Alteromonas macleodii str. 'Black Sea 11'] (AFT77553.1) | 39/61(301) | UDP-glucose 4-epimerase |
| 4 | *GT1* | 4237..5376 | 33.07 | Glycos_transf_1; PF00534.16; E value=6.8e-10 | glycosyl transferase group 1 [Desulfomicrobium baculatum DSM 4028] (ACU91360.1) | 37/52(379) | glycosyl transferase |
| 5 | *wzy* | 5379..6530 | 31.25 | EpsG; PF14897.2; E value=1e-33 | Wzy [Acinetobacter baumannii] (AHB32799.1) | 33/49(383) | O antigen polymerase |
| 6 | *GT2* | 6532..7647 | 32.8 | Glyphos_transf; PF04464.10; E value=5.7e-37 | CDP-glycerol:poly(glycerophosphate) glycerophosphotransferase [Escherichia coli] (AJE24488.1) | 57/72(371) | CDP-glycerol:poly(glycerophosphate) glycerophosphotransferase |
| 7 | *GT3* | 7649..8920 | 32.39 | Glycos_transf_1; PF00534.16; E value=2.1e-19 | putative glycosyltransferase [Escherichia coli] (BAQ01295.1) | 57/75(423) | putative glycosyltransferase |
| 8 | *gmd* | 8913..10037 | 43.82 | GDP_Man_Dehyd; PF16363.1; E value=2.8e-159 | GDP-mannose 4,6-dehydratase [Escherichia albertii] (WP_105228360.1) | 62/96(374) | GDP-mannose 4,6-dehydratase |
| 9 | *fcl* | 10041..11006 | 42.34 | Epimerase; PF01370.17; E value=7.6e-73 | GDP-L-fucose synthase [Cedecea lapagei] (WP_100779633.1) | 95/98(321) | GDP-L-fucose synthase |
| 10 | *gmm* | 11009..11470 | 37.45 | NUDIX; PF00293.24; E value=9.8e-16 | GDP-mannose mannosyl hydrolase [Cedecea lapagei] (WP_100779634.1) | 90/94(153) | GDP-mannose mannosyl hydrolase |
| 11 | *manC* | 11476..12882 | 45.84 | NTP_transferase; PF00483.19; E value=1e-71 | mannose-1-phosphate guanylyltransferase/mannose-6-phosphate isomerase [Escherichia coli] (WP_032314591.1) | 84/93(468) | Mannose-1-phosphate guanylyltransferase |
| 12 | *GT4* | 12882..13628 | 41.37 | Glycos_transf_2; PF00535.22; E value=4.3e-26 | glycosyltransferase [Escherichia coli](WP_089586530.1) | 82/92(248) | putative glycosyltransferase |
| 13 | *manB* | 13633..15054 | 45.29 | PGM_PMM_I; PF02878.12; E value=5e-25 | phosphomannomutase [Cedecea lapagei] (WP_100779637.1) | 90/93(473) | phosphomannomutase |

| *E. cloacae* O27 | | | | | | | |
| --- | --- | --- | --- | --- | --- | --- | --- |
| Orf no. | Gene name | Position of gene | G+C content(%) | Conserved domain(s) | Similar protein(s), strain(s) (Genbank accession No.) | %Identical/%Similar (total No. of aa) | Putative function of protein |
| 1 | *wbpK* | 1281..2225 | 44.76 | Epimerase; PF01370.17; E value=1e-28 | NAD-dependent epimerase/dehydratase [Enterobacter sp. Ag1] (EJF32346.1) | 60/78(314) | NAD-dependent epimerase/dehydratase |
| 2 | *wbpL* | 2225..3268 | 47.22 | Glycos_transf_4; PF00953.17; E value=5.2e-28 | polyprenol phosphate:N-acetyl-hexosamine 1-phosphate transferase [Yersinia frederiksenii] (CNF58510.1) | 53/70(347) | polyprenol phosphate:N-acetyl-hexosamine 1-phosphate transferase |
| 3 | *AT* | 3261..3821 | 47.06 | Hexapep; PF00132.20; E value=3e-08 | sugar O-acyltransferase [Enterobacter cloacae complex sp. 35669] (WP_045260407.1) | 99/99(186) | UDP-N-acetylbacillosamine N-acetyltransferase |
| 4 | *wbpM* | 3860..5746 | 47.27 | Polysacc_synt_2; PF02719.11; E value=1.4e-127 | nucleoside-diphosphate sugar epimerase [Enterobacter cloacae complex sp. 35669] (KJN34375.1) | 98/98(628) | nucleoside-diphosphate sugarepimerase/dehydratase |
| 5 | *rmlB* | 5849..6934 | 49.63 | GDP_Man_Dehyd; PF16363.1; E value=3.9e-99 | dTDP-glucose 4,6-dehydratase [Enterobacter cloacae] (WP_048992939.1) | 92/95(361) | dTDP-glucose 4,6-dehydratase |
| 6 | *rmlD* | 6934..7833 | 52.11 | RmlD_sub_bind; PF04321.13; E value=5.2e-107 | dTDP-4-dehydrorhamnose reductase [Enterobacter cloacae] (WP_063162896.1) | 99/99(299) | dTDP-4-dehydrorhamnose reductase |
| 7 | *rmlA* | 7886..8767 | 43.65 | NTP_transferase; PF00483.19; E value=3.7e-74 | glucose-1-phosphate thymidylyltransferase RfbA [Enterobacter cloacae] (WP_063923190.1) | 96/98(293) | glucose-1-phosphate thymidylyltransferase |
| 8 | *GT1* | 8898..9704 | 38.04 | Glycos_transf_2; PF00535.22; E value=1.4e-21 | putative glycosyltransferase [Escherichia coli] (BAQ00849.1) | 52/70(268) | putative glycosyltransferase |
| 9 | *rmlC* | 9745..10293 | 36.07 | dTDP_sugar_isom; PF00908.13; E value=5.2e-78 | dTDP-4-dehydrorhamnose 3,5-epimerase [Escherichia coli] (WP_044720521.1) | 66/82(182) | dTDP-4-dehydrorhamnose 3,5-epimerase |
| 10 | *fdtA* | 10293..10688 | 36.87 | FdtA; PF05523.7; E value=2.5e-54 | FdtA [Cronobacter muytjensii] (AEH27510.1) | 84/91(131) | TDP-4-oxo-6-deoxy-alpha-D-glucose-3, 4-oxoisomerase |
| 11 | *fdtB* | 10705..11808 | 38.04 | DegT_DnrJ_EryC1; PF01041.13; E value=1.9e-111 | DegT/DnrJ/EryC1/StrS family aminotransferase [Citrobacter freundii] (WP_003842215.1) | 86/92(367) | dTDP-3-amino-3,6-dideoxy-alpha-D-galactopyranose transaminase |
| 12 | *wzx* | 11832..13055 | 38.4 | Polysacc_synt; PF01943.13; E value=3.4e-13 | O-antigen flippase [Cronobacter dublinensis] (WP_105687502.1) | 51/73(407) | O-antigen flippase |
| 13 | *wzy* | 13068..14366 | 32.56 |  | oligosaccharide repeat unit polymerase [Escherichia coli] (WP_072645697.1) | 23/42(432) | polymerase |
| 14 | *GT2* | 14376..15278 | 31.67 | Glycos_transf_2; PF00535.22; E value=2e-21 | glycosyl transferase [Shewanella violacea] (WP_013050767.1) | 40/58(300) | glycosyl transferase |
| 15 | *GT3* | 15271..16167 | 33.33 | Glycos_transf_2; PF00535.22; E value=1.7e-11 | glycosyltransferase family 2 protein [Serratia sp. Leaf51] (WP_056769570.1) | 61/73(298) | glycosyl transferase |
| 16 | *fdtC* | complement (16263..16718) | 36.84 | Hexapep; PF00132.20; E value=2.9e-22 | N-acetyltransferase [Vibrio cholerae] (WP_032482284.1) | 75/88(151) | dTDP-3-amino-3,6-dideoxy-alpha-D-galactopyranose 3-N-acetyltransferase |

| *E. cloacae* O30 | | | | | | | |
| --- | --- | --- | --- | --- | --- | --- | --- |
| Orf no. | Gene name | Position of gene | G+C content(%) | Conserved domain(s) | Similar protein(s), strain(s) (Genbank accession No.) | %Identical/%Similar (total No. of aa) | Putative function of protein |
| 1 | *rmlB* | 1257..2342 | 52.03 | GDP_Man_Dehyd; PF16363.1; E value=4.1e-98 | dTDP-glucose 4,6-dehydratase [Enterobacter cloacae] (WP_063137675.1) | 98/98(361) | dTDP-glucose 4,6-dehydratase |
| 2 | *rmlD* | 2342..3241 | 50.89 | RmlD_sub_bind; PF04321.13; E value=3.7e-108 | dTDP-4-dehydrorhamnose reductase [Enterobacter cloacae] (WP_095908021.1) | 97/98(299) | dTDP-4-dehydrorhamnose reductase |
| 3 | *rmlA* | 3294..4172 | 48.12 | NTP_transferase; PF00483.19; E value=1.8e-73 | glucose-1-phosphate thymidylyltransferase RfbA [Enterobacter cloacae] (WP_086581292.1) | 98/98(292) | Glucose-1-phosphate thymidylyltransferase |
| 4 | *rmlC* | 4176..4736 | 37.79 | dTDP_sugar_isom; PF00908.13; E value=2.4e-73 | dTDP-4-dehydrorhamnose 3,5-epimerase [Enterobacter cloacae] (WP_095908019.1) | 94/97(186) | dTDP-4-dehydrorhamnose 3,5-epimerase |
| 5 | *GT1* | 4733..5653 | 33.12 | Glycos_transf_2; PF00535.22; E value=1.7e-36 | glycosyl transferase family 2 [Escherichia coli] (WP_106485890.1) | 60/76(306) | Putative glycosyltransferase EpsE |
| 6 | *GT2* | 5654..6700 | 33.72 | Glycos_transf_1; PF00534.16; E value=2.5e-19 | glycosyltransferase family 1 protein [Xenorhabdus bovienii] (WP_038197794.1) | 46/66(348) | glycosyltransferase |
| 7 | *GT3* | 6697..7452 | 32.94 | Glyco_transf_7C; PF02709.10; E value=2.5e-06 | glycosyltransferase family 2 protein [Escherichia coli] (WP_073278741.1) | 59/75(251) | Rhamnosyltransferase WbbL |
| 8 | *wzx* | 7445..8665 | 32.27 | Polysacc_synt_3; PF13440.2; E value=9e-17 | O-antigen flippase [Escherichia coli] (AIG62382.1) | 59/76(406) | flippase |
| 9 | *orf9* | 8637..9659 | 29.42 | ATP-grasp_4; PF13535.2; E value=1.6e-09 | hypothetical protein [Enterobacter cloacae] (WP_095908014.1) | 95/97(340) | hypothetical protein |
| 10 | *GT4* | 9634..10692 | 33.71 | Glycos_transf_1; PF00534.16; E value=1.6e-14 | glycosyltransferase family 1 protein [Vibrio breoganii] (WP_102446408.1) | 44/61(352) | glycosyl transferases |
| 11 | *wzy* | 10689..11828 | 30.44 | O-antigen_lig; PF13425.2; E value=8.9e-13 | O48 family O-antigen polymerase [Escherichia coli] (WP_069915474.1) | 51/72(379) | O-antigen polymerase |
| 12 | *galE* | 11832..12842 | 35.41 | GDP_Man_Dehyd; PF16363.1; E value=3.2e-56 | UDP-glucose 4-epimerase GalE [Escherichia coli] (WP_073278729.1) | 65/78(336) | UDP-glucose 4-epimerase |
