# Supplementary material for: Genetic Characterization of the O-Antigen and Development of a Molecular Serotyping Scheme for Enterobacter cloacae
Source: Front Microbiol. 2020 Apr 28;11:727. doi: 10.3389/fmicb.2020.00727 (PMC7198725; doi:10.3389/fmicb.2020.00727)
Supplement: Supplementary file 5 [file Table_2.doc]

Supplementary Table 2. Putative serotype allocation of *E. cloacae* strains from Genbank

| Putative serotype | Strian(s) |
| --- | --- |
| temp1 | e2054, e332, e483, DG6 |
| temp2 | ND22 |
| temp3 | e1130, e1873, e2473, e552, e627, e629, e716 |
| temp4 | AR_0093 |
| temp5 | e1589, e2005, e2048, e2049, e2127, e294 |
| temp6 | e554, e804, e1267, e798 |
| temp7 | e1202, e1347, e1690, e1983, e590, e812 |
| temp8 | e1198, e1227, e1272, e1337, e1547, e247, e272, e289, e301, e559, e633, e894, e965, NCTC13406 |
| temp9 | B2 |
| temp10 | e1422 |
| temp11 | CAPREX_E2-2, CAPREX_E7, CIDEIMsCOL10 |
| temp12 | UCI102 |
| temp13 | GGT036 |
| temp14 | SDM, HB_Z_2347 |
| temp15 | SUBG009 |
| temp16 | e1942, e1635, e638 |
| temp17 | e1265 |
| temp18 | GN02616 |
| temp19 | e929 |
| temp20 | e2161 |
| temp21 | e458, e659, e1026 |
| temp22 | e1718, e1719 |
| temp23 | DS11005, DS18454, DS25697, DS31253, DU20756 |
| temp24 | DS17594 |
| temp25 | SMART_1312 |
| temp26 | e1713, e765 |
| temp27 | SMART_855 |
| temp28 | e2032 |
| temp29 | 6kgm |
| temp30 | e1617, e864 |
| temp31 | e264 |
| temp32 | e558 |
| temp33 | e1236, e698 |
| temp34 | DS9199 |
| temp35 | GN05902 |
| temp36 | e1316, e319 |
| temp37 | e811 |
| temp38 | GN02174 |
| temp39 | AM16-28 |
| temp40 | NCTC11571 |
| temp41 | e2760, e1343 |
| temp42 | e773, e1252 |
| temp43 | e1527 |
| temp44 | e977 |
| temp45 | EC_073 |
| temp46 | e456 |
| temp47 | AR_0154 |
| temp48 | e362 |
| temp49 | e917 |
| temp50 | e1323 |
| temp51 | DS11861, 170, DS8140 |
| temp52 | SMART_901, INSali2 |
| temp53 | e915 |
| temp54 | amazonensis, SMART_313 |
| temp55 | AR_0050 |
